# Caspase-4/11 promotes hyperlipidemia and chronic kidney disease–accelerated vascular inflammation by enhancing trained immunity

**DOI:** 10.1172/jci.insight.177229

**Published:** 2024-07-18

**Authors:** Yu Sun, Yifan Lu, Lu Liu, Fatma Saaoud, Ying Shao, Keman Xu, Charles Drummer, Ramon Cueto, Huimin Shan, Xiaohua Jiang, Huaqing Zhao, Hong Wang, Xiaofeng Yang

**Affiliations:** 1 Lemole Center for Integrated Lymphatics and Vascular Research, Department of Cardiovascular Sciences, Lewis Katz School of Medicine at Temple University, Philadelphia, Pennsylvania, USA.; 2 Department of Molecular Biology, Princeton University, Princeton, New Jersey, USA.; 3 Centers of Metabolic Disease Research and Thrombosis Research Center, Department of Cardiovascular Sciences, and; 4 Center for Biostatistics and Epidemiology, Department of Biomedical Education and Data Sciences, Lewis Katz School of Medicine at Temple University, Philadelphia, Pennsylvania, USA.

**Keywords:** Inflammation, Vascular biology, Chronic kidney disease, Vasculitis

## Abstract

To determine whether hyperlipidemia and chronic kidney disease (CKD) have a synergy in accelerating vascular inflammation via trained immunity (TI), we performed aortic pathological analysis and RNA-Seq of high-fat diet–fed (HFD-fed) 5/6 nephrectomy CKD (HFD+CKD) mice. We made the following findings: (a) HFD+CKD increased aortic cytosolic LPS levels, caspase-11 (*CASP11*) activation, and 998 gene expressions of TI pathways in the aorta (first-tier TI mechanism); (b) *CASP11^–/–^* decreased aortic neointima hyperplasia, aortic recruitment of macrophages, and casp11–gasdermin D–mediated cytokine secretion; (c) *CASP11^–/–^* decreased N-terminal gasdermin D (N-GSDMD) membrane expression on aortic endothelial cells and aortic *IL-1B* levels; (d) LPS transfection into human aortic endothelial cells resulted in *CASP4* (human)/*CASP11* (mouse) activation and increased N-GSDMD membrane expression; and (e) *IL-1B* served as the *s*econd-tier mechanism underlying HFD+CKD-promoted TI. Taken together, hyperlipidemia and CKD accelerated vascular inflammation by promoting 2-tier trained immunity.

## Abstract 2

hyperlipidemia and chronic kidney disease have a synergy in accelerating vascular inflammation via trained immunity

## Introduction

Chronic kidney disease (CKD) is a common inflammatory disease affecting > 15% of the adult population (1). In total, 50% of all patients with advanced/end stages (stages 4 and 5) of CKD have cardiovascular disease (CVD), and CVD accounts for 50% of all deaths in patients with advanced CKD (2). Endotoxemia generated from Gram-negative bacteria, detected in as many as 53% of patients with CKD (3), is a novel and significant risk factor for systemic inflammation and CVD in patients with CKD (4) and is treated with hemodialysis (5). In addition, 76.2% of male patients with CKD were infected with urinary tract infections, and 94% were gram-negative bacteria (6). A significant question remains poorly characterized on how multiple disease risk factors such as CVD, CKD, and endotoxemia interplay to accelerate vascular inflammation.

Vascular inflammation contributes significantly to the atherosclerotic onset and development of its complications (7). A long list of findings from rodent models and the Canakinumab Anti-inflammatory Thrombosis Outcomes Study (CANTOS) demonstrated that the inhibition of proinflammatory *IL1B* and other proinflammatory cytokines and regulators reduce the atherosclerotic burden in CVDs (8). New progress from our and others’ teams allowed us to propose concepts: endothelial cells (ECs) are innate immune cells (9) that highlight the innate immune functions and innate immune memory (trained immunity [TI]) of ECs (10), similar to prototypic innate immune cells such as monocytes (11) and macrophages (12). Uremic toxins (UTs) related to CKD are danger-associated molecular patterns (DAMPs) (13) that activate the sensor caspase-1 (*Casp1*)/inflammasome and promote neointimal hyperplasia (14). Furthermore, CKD and end-stage renal disease (ESRD) upregulate secretomic genes and promote inflammation (15). However, how gram-negative bacteria–derived endotoxins such as LPS in CKD and CVD pathologies enter aortic ECs and trigger vascular inflammation remains poorly characterized.

Three proinflammatory caspases, including *Casp1*, *Casp4* (human)/*Casp11* (mouse), and *Casp5*, are involved in sensing DAMPs and initiating inflammation and inflammatory cell death (pyroptosis). Two major inflammasome pathways have been characterized: canonical and noncanonical inflammasomes. Canonical inflammasomes are the protein complexes for activation of *Casp1* (16, 17). The noncanonical inflammasomes are assembled for activation of *Casp11* by binding with intracellular gram-negative bacteria endotoxin LPS — potentially guanylate-binding proteins (GBPs) (16). *Casp4/11* plays significant roles in promoting defense against gram-negative bacteria that escape the phagosome and invade the cytosol (18) by clearing invaded bacterial pathogens (19), alerting neighboring cells, initiating pyroptosis (20), and contributing to endothelial pyroptosis and lung pathologies (21). However, compared with *Casp1* and canonical inflammasomes (17), the roles of noncanonical inflammasomes and *Casp4/11* in vascular diseases (22) remain much less characterized.

As we and others reported (23), innate immune cells, including ECs (9), can develop an exacerbated immunologic response and long-term inflammatory phenotype following brief exposure to endogenous or exogenous PAMPs/DAMPs (7). This persistent hyperactivation of the innate immune system is termed TI or innate immune memory and can contribute to the pathophysiology of atherosclerosis (24). TI in vascular cells is functional for enhancing inflammation effectiveness and transitioning to chronic inflammation. However, 2 important questions remained: (a) do CKD and CVD accelerate vascular inflammation via enhancing TI in aortic vascular cells, and (b) is the noncanonical inflammasome signaling (*CASP4/11*-*GSDMD*-*IL1B*) the underlying molecular mechanisms of TI in CKD and CVD?

To address those important questions, we used a 5/6 nephrectomy CKD mouse model fed with a high-fat diet (HFD) for 10 weeks to establish a HFD+CKD mouse model. We also performed aortic RNA-Seq transcriptomic analysis, single-cell RNA-Seq data analysis, mitochondrial reactive oxygen species (mitoROS) and mitoROS inhibitor mitoTEMPO functional assays, and gasdermin D (*GSDMD*) cleavage peptide inhibitor in combination with extensive flow cytometry and histopathological analyses. Our results have provided insights into the role of hyperlipidemia and CKD in accelerating vascular inflammation via a 2-step TI mechanism and therapeutic targets for treating CKD, gram-negative bacterial infections, CVD, inflammations, immune diseases, transplantation, aging, and cancers.

## Results

### HFD+CKD increases plasma LDL-VLDL, aortic cytosolic LPS levels, Casp11 activation, and 998 gene expressions with TI pathways in the aorta.

**Figure 1:**
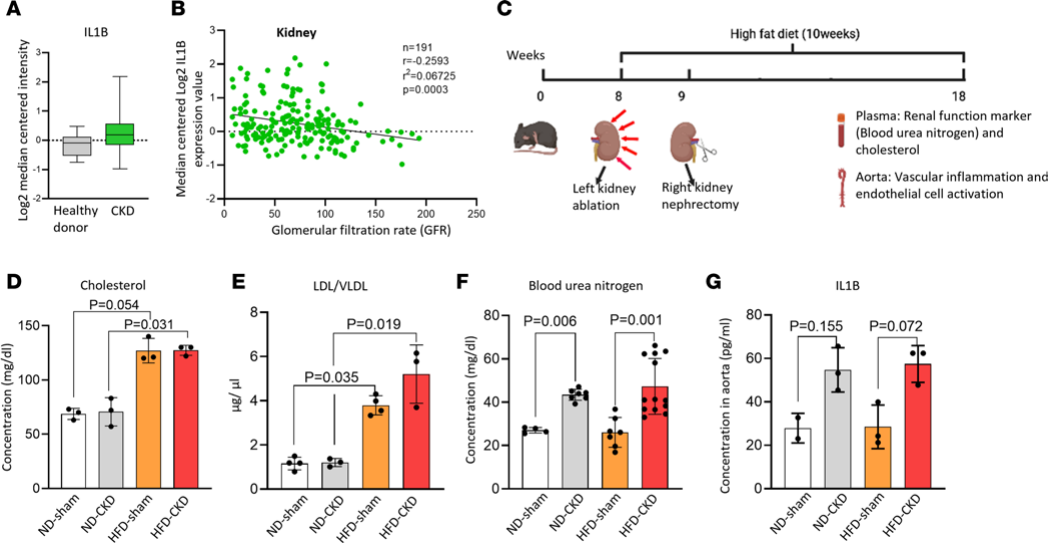
*IL1B* is positively correlated with chronic kidney disease (CKD) progression. (**A**) The expression of *IL1B* was increased in the glomeruli of patients with CKD. (**B**) *IL1B* expression in kidneys was inversely correlated with glomerular filtration rates (GFR) (Pearson analysis). The data were extracted from human Glomeruli samples (199 samples) in the NephroSeq database. (**C**) High-fat diet-fed (HFD) 5/6 nephrectomy mouse model of CKD with sham controls and normal chow diet (ND) controls. (**D** and **E**) HFD resulted in hyperlipidemia/dyslipidemia but did not exacerbate *IL1B* levels in the kidney or impair kidney function. (**D**) Cholesterol levels in the plasma of HFD+CKD, HFD-sham, ND-CKD, and ND-sham mice (<100 was considered normal by The Jackson Laboratory). (**E**) LDL/VLDL levels in the plasma of HFD+CKD, HFD-sham, ND-CKD, and ND-sham mice (*n* = 3–4 per group). (**F**) Blood urea nitrogen (BUN) levels in the plasma of HFD+CKD, HFD-sham, ND-CKD, and ND-sham mice. BUN < 24 was considered normal based on Mayo Clinic criteria (*n* = 5 in ND-sham, *n* = 7 in ND-CKD and HFD-sham, *n* = 13 in HFD+CKD). (**G**) *IL1B* levels in the aorta of HFD+CKD, HFD-sham, ND-CKD, and ND-sham. Two-tailed Student’s *t* test was used in **A**; Pearson correlations were used in **B**. The Kruskal-Wallis test with Benjamini and Hochberg multiple-comparison method was used to control the overall FDR of 5% (**D**–**G**).

We first examined whether the expression of *IL1B*, a substrate of proinflammatory *CASP1*, is correlated with the progression of CKD by searching for clinically relevant data in the Nephroseq database. *IL1B* expression levels were increased in the kidneys of patients with CKD compared with healthy controls (Figure 1A). In addition, increased *IL1B* expressions in the kidneys of 191 patients with CKD were inversely correlated with the glomerular filtration rate (GFR) (Figure 1B), suggesting that *IL1B* secretion, activities of *CASP1*-inflammasome, and *CASP4/11*-noncanonical inflammasome may be increased in CKD and are similar to the cleavage of *CASP1* and *CASP11* reported in the rat model of hyperuricemic nephropathy (25), roles of *NLRP3* inflammasome in various kidney diseases (26), and animal models of kidney diseases (27).

Hyperlipidemia and dyslipidemia have been reported to be clinically associated with systemic inflammation in patients with CKD (Table 1) (28–33). However, a recent paper also reported negative correlations of total, non–high-density lipoprotein (non-HDL), and low-density lipoprotein (LDL) cholesterol with a panel of UTs based on a cohort of 611 patients with kidney failure (34). To solve this controversial issue in CKD systemic inflammation and determine novel mechanisms underlying how hyperlipidemia and CKD UTs synergistically accelerate vascular inflammation, we established a 10-week HFD+CKD model, as we reported (14) (Figure 1C). As shown in Figure 1, D–G, HFD+CKD significantly increased levels of cholesterol and LDL/VLDL compared with normal chow diet (ND) sham (ND-sham) and ND-CKD. However, blood urea nitrogen (BUN) levels and *IL1B* levels in HFD+CKD were equivalent to those of ND-CKD, suggesting that kidney dysfunction was not further aggravated in the HFD+CKD group in the experimental setting (Supplemental Figure 1; supplemental material available online with this article; https://doi.org/10.1172/jci.insight.177229DS1).

**Figure 2:**
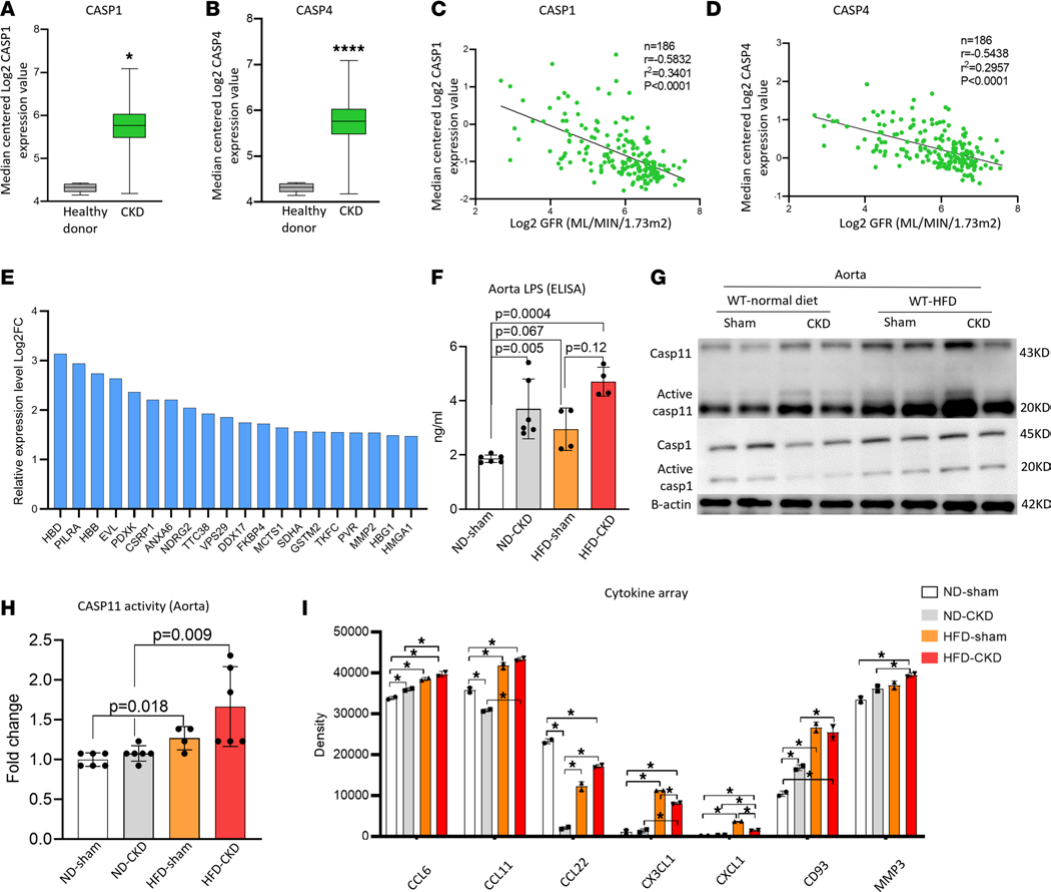
The expression and activity of caspase-4 are positively associated with the progression of CKD-accelerated vascular inflammation. (**A**) Casppase-1 (casp1) expression in the kidneys of patients with CKD compared with that of healthy donors. (**B**) Caspase-4 (*CASP4*) expression in the kidneys of patients with CKD compared with that of healthy donors. (**C** and **D**) *CASP1* and *CASP4* were negatively correlated with GFR (Pearson analysis). (**E**) The top 20 CASP4/Gasdermine D–related (GSDMD-related) secretion genes were significantly increased in patients with CKD (GSE66494). (**F**) The LPS level in the aorta of HFD+CKD, HFD-Sham, ND-CKD, and ND-Sham mice was detected by an ELISA kit (*n* = 4–6). (**G**) Casp11 activation scales in the HFD+CKD aorta were higher than those of *CASP1*. Western blot analysis of WT mouse aortic tissue for *CASP1* and casp11. (**H**) Casp11 activities in the aorta of HFD+CKD, HFD-sham, ND-CKD, and ND-sham were detected by the casp4 activity assay. In total, 50 μg protein from each sample was used to detect casp11 activities (*n* = 4–6). (**I**) The proinflammatory cytokines in the plasma of HFD+CKD, HFD-sham, ND-CKD, and ND-sham were analyzed by cytokine array. Each sample was pooled from 3 mice in each group (*n* = 3). ImageJ was used to quantify the bands, and the significantly changed proteins were indicated. Two-tailed Student’s *t* test was used in **A** and **B**. Pearson correlations were used in **C** and **D**. The Kruskal-Wallis test with Benjamini and Hochberg multiple-comparison method was used to control the overall FDR of 5% (**F**, **H**, and **I**). *P < 0.05.

We reported that UTs-promoted neointima hyperplasia in the carotid artery was significantly inhibited in *CASP1/CASP11^–/–^* mice (14). To further determine whether *CASP1* and *CASP4/11* pathways are pathophysiologically relevant to CKD, we found that the expression levels of *CASP1* and *CASP4* were significantly increased in the kidneys of patients with CKD (Figure 2, A and B). In addition, *CASP1* and *CASP4* expression levels were negatively correlated with GFR in patients with CKD (Figure 2, C and D). Moreover, our database mining analysis showed that 20 *CASP4/11-GSDMD* secretome genes (35, 36) were highly expressed in the renal biopsies obtained from 48 patients with CKD (37) (Figure 2E). As reported, LPS is increased in the serum of patients with CKD (Table 2), and casp4/11 detects intracellular bacterial endotoxin LPS stimulation (4, 18, 38–44). However, an important consideration remains: whether cytosolic LPS is increased in CKD vessels. We found that cytosolic LPS levels were significantly increased in the aortas of CKD and HFD+CKD mice (Figure 2F), and LPS in HFD+CKD mice was higher than that in CKD mice, suggesting that HFD presumably further promotes LPS endocytosis. We then found that the active *CASP11* was increased in HFD+CKD aortas compared with ND-sham, ND-CKD, and HFD-sham (Figure 2G), suggesting that HFD promotes aortic *CASP11* activation higher than does CKD alone. However, Western blots did not show a marked increase in active *CASP1* in the aorta in these groups. Of note, it was reported that enteropathogenic endotoxin-generating E. *coli* use NleA to inhibit *NLRP3* inflammasome activation (45), implying that this mechanism may result in *CASP4/11* activation but inhibits *CASP1* activation. To further confirm the results that HFD+CKD–increased *CASP11* activation in aortas compared with the other groups, we used the *CASP11* activity assay and found that *CASP11* activity in HFD+CKD aorta was higher than that of CKD and HFD-sham (Figure 2H). To further determine whether any of the mass spectrometry–identified *CASP4/GSDMD* secretomes, including cytokines and chemokines (36), are increased in HFD+CKD, we measured plasma cytokines and chemokines using a mouse cytokine array (Supplemental Figure 2). HFD+CKD significantly upregulated 7 cytokines and chemokines (Figure 2I). Among the 7 upregulated plasma cytokines and chemokines, *CCL22* was 1 of the *CASP4/GSDMD* secretomic cytokines and chemokines. Taken together, our results have demonstrated that hyperlipidemia and CKD significantly increase the plasma LDL-VLDL level and cytosolic LPS levels in the aorta; activate *CASP4/11* more than *CASP1*; and increase 7 secretome cytokines and chemokines in plasma. Of note, potassium (K^+^) efflux also contributes to inflammasome activation. We examined 79 K^+^ channel genes and found only 1 gene upregulated in the HFD+CKD condition (Supplemental Figure 4F).

### Casp11^–/–^ decreases aortic neointima hyperplasia, recruitment of monocytes and macrophages into the aorta, and secretion of the Casp11–GSDMD secretome cytokine CCL22 in plasma.

**Figure 3:**
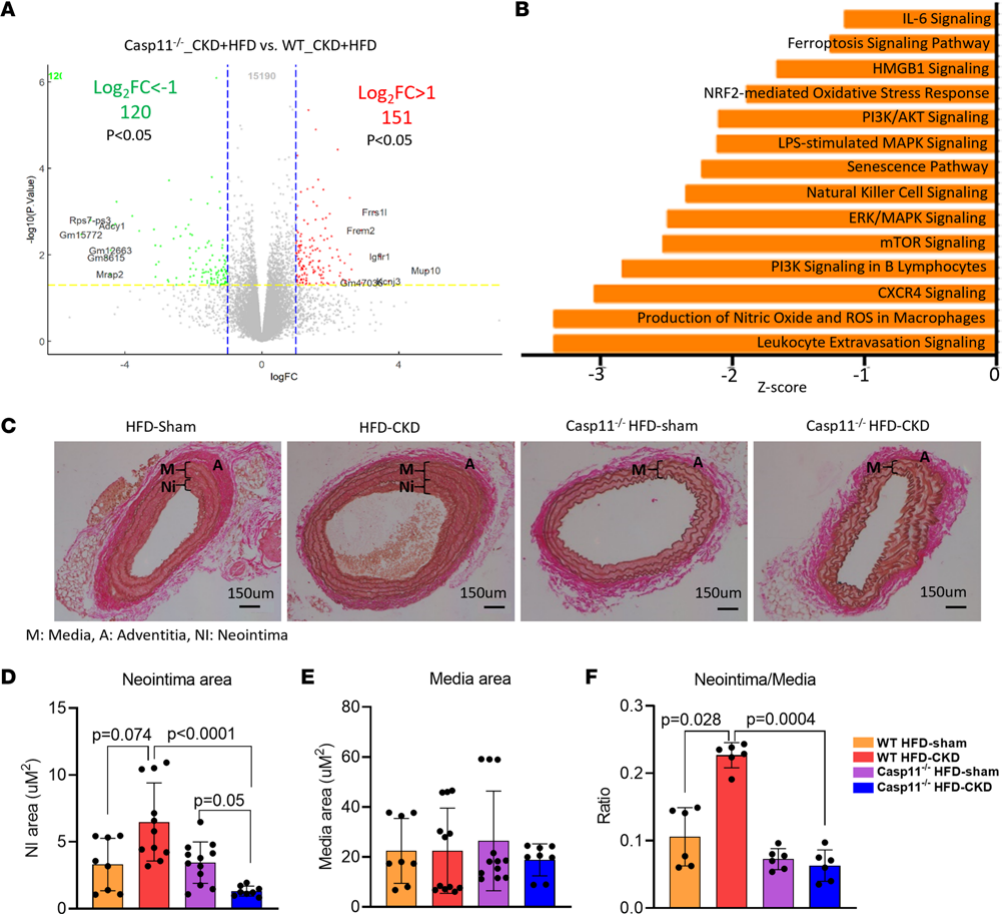
Caspase-11 deficiency decrease the formation of neointima in aortas of HFD+CKD mice. (**A**) Volcano plot analysis of bulk RNA-Seq data showed the significantly modulated genes in *casp11*^–/–^ CKD+HFD compared with WT CKD+HFD. Green number is original software generated data indicating 120 downregulated genes. (**B**) Ingenuity Pathway Analysis (IPA) of upregulated pathways in *casp11*^–/–^ CKD+HFD upregulated genes compared with WT CKD+HFD upregulated genes. (*P* < 0.05, *Z* score < –1). (**C**) The Verhoeff–van Gieson stain of mouse aorta showed that HFD+CKD increased neointima area and the ratios of neointima/media in WT aortas, which were significantly suppressed in *casp11*^–/–^ HFD+CKD aortas. (**D**–**F**) The quantifications of neointima, media, and neointima/media. The Kruskal-Wallis test with Benjamini and Hochberg multiple-comparison method was used to control the overall FDR of 5% (**D**–**F**).

We performed RNA-Seq analysis to examine the *CASP11* deficiency–regulated proinflammatory transcriptome. Results showed that *CASP11^–/–^* significantly downregulated 120 genes and decreased 14 inflammatory pathways, including *IL6* signaling pathway, ferroptosis signaling pathway, *HMGB1* signaling pathway, *NRF2*-mediated oxidative stress response, PI3K/Akt signaling pathway, LPS-stimulated MAPK signaling pathway, senescence pathway, NK cell signaling pathway, ERK/MAPK signaling pathway, mTOR signaling pathway, PI3K signaling in B lymphocytes, CXCR4 signaling pathway, production of nitric oxide and ROS in macrophages, and leukocyte extravasation signaling pathway (Figure 3, A and B). To verify the transcriptomic data, we performed histochemical staining of the carotid artery to examine the neointima and media layers. Our data show that HFD+CKD increased neointima area with no significant changes in media area, leading to an increase in the ratio of neointima/media in WT aortas, and this ratio was significantly suppressed in HFD+CKD *CASP11^–/–^* aortas (Figure 3, C–F), suggesting that *CASP11* deficiency decreased HFD+CKD-induced neointima hyperplasia. Of note, since WT C57BL/6 mice with 10-week HFD are not an atherogenic mouse model (46), atherosclerosis was not examined.

**Figure 4:**
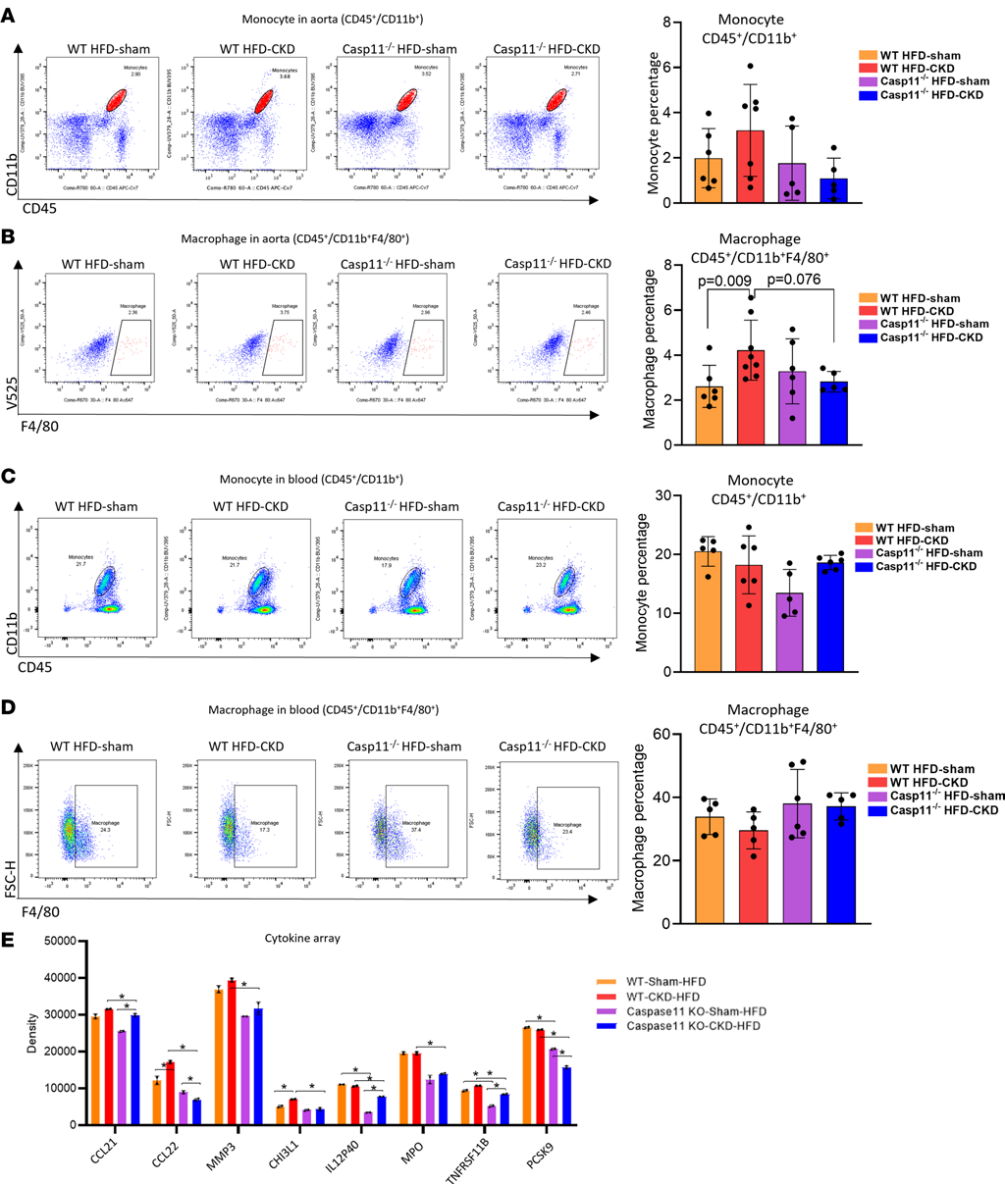
Caspase-11 deficiency inhibits inflammatory cell infiltration into the aortas of HFD+CKD mice. (**A** and **B**) Flow cytometry analysis demonstrated that HFD+CKD increased the recruitment of CD45^+^CD11b^+^ monocytes and CD45^+^CD11b^+^F4/80^+^ macrophages into the WT aorta, which were significantly suppressed in HFD+CKD *casp11*^–/–^ aortas. (**C** and **D**) Flow cytometry analysis demonstrated that *casp11*^–/–^ decreased blood CD45^+^CD11b^+^ monocytes in HFD-sham mice compared with WT HFD-sham mice; and HFD+CKD mice did not significantly change blood CD45^+^CD11b^+^ monocytes and CD45^+^CD11b^+^F4/80^+^ macrophages in HFD+CKD mice (*n* = 6–8). Flow cytometry analysis showed the infiltrated monocytes (CD11b^+^CD45^+^) and macrophages (CD11b^+^CD45^+^F4/80^+^) in the blood of WT and *casp11*^–/–^ HFD+CKD and HFD-Sham mice. (**E**) Cytokine array showed that *casp11*^–/–^ decreased HFD+CKD-induced chemokines and cytokines, including CCL2 and CCL22, MMP-3, chinitianase 3-like 1 (CHIL3L1), IL-12p40, myeloperoxidase, TNFRSF11b, and PCSK9 in plasma. Each sample was pooled from 3 mice in each group (*n* = 3). ImageJ was used to quantify the bands, and the significantly changed proteins were indicated. The Kruskal-Wallis test with Benjamini and Hochberg multiple-comparison method was used to control the overall FDR of 5% (**A**–**E**).

In addition, we performed flow cytometric analysis to examine whether monocyte and macrophage recruitment into the aortas is decreased in *CASP11^–/–^*. As shown in Figure 4, A and B, HFD+CKD increased the recruitment of CD45^+^CD11b^+^ monocytes and CD45^+^CD11b^+^F4/80^+^ macrophages into WT aortas, which were suppressed in HFD+CKD *CASP11*^–/–^ aortas. However, *CASP11*^–/–^ decreased blood CD45^+^CD11b^+^ monocytes in HFD sham mice compared with WT HFD sham mice, and *CASP11*^–/–^ did not significantly change blood CD45^+^CD11b^+^ monocytes or CD45^+^CD11b^+^F4/80^+^ macrophages in HFD+CKD mice (Figure 4, C and D), suggesting that HFD+CKD activates *CASP11* and promotes monocyte and macrophage migration into the aorta rather than increasing the generation of monocytes and macrophages in the blood. Furthermore, we performed cytokine array analysis to examine cytokines and chemokines in the plasma of CKD+HFD and sham-HFD of both WT and *CASP11*^–/–^ mice (Figure 4E). The data show that WT CKD+HFD significantly increased chemokine *CCL22* and *CHI3L1* in the blood compared with WT sham-HFD, and *CASP11* deficiency decreased 8 chemokines and cytokines in the blood: *CCL21*, *CCL22*, *MMP-3*, *CHI3L1*, *IL12B*, *MPO*, *TNFRSF11B*, and *PCSK9*. These results demonstrate that WT CKD+HFD increased neointima hyperplasia and aortic macrophage recruitment compared with WT sham-HFD; however, *CASP11* deficiency decreased recruitment of aortic monocytes and macrophages and reduced plasma cytokines and chemokines. Of note, since it has been reported that loss-of-function variants of *PCSK9* are associated with low circulating levels of LDL cholesterol (LDL-C) and a reduced risk of coronary artery disease (47), our results suggest a function of *CASP11* in promoting LCL-C by enhancing *PCSK9*. Future work is warranted to determine whether decreased *PCSK9* contributes to increased plasma LDL-VLDL in CKD+HFD mice. In addition, the Venn diagram analysis (Supplemental Figure 4, D and E) indicates that *Casp11* deficiency may alter adipocyte-like vascular smooth muscle cells via the upregulation of iodothyronine deiodinase 2 (*DIO2*) in the mouse aorta under conditions of HFD and CKD.

### Casp11^–/–^ decreases N-terminal GSDMD expression on the mouse aortic CD45^–^CD31^+^ EC membrane and reduces mouse aortic IL1B levels.

**Figure 5:**
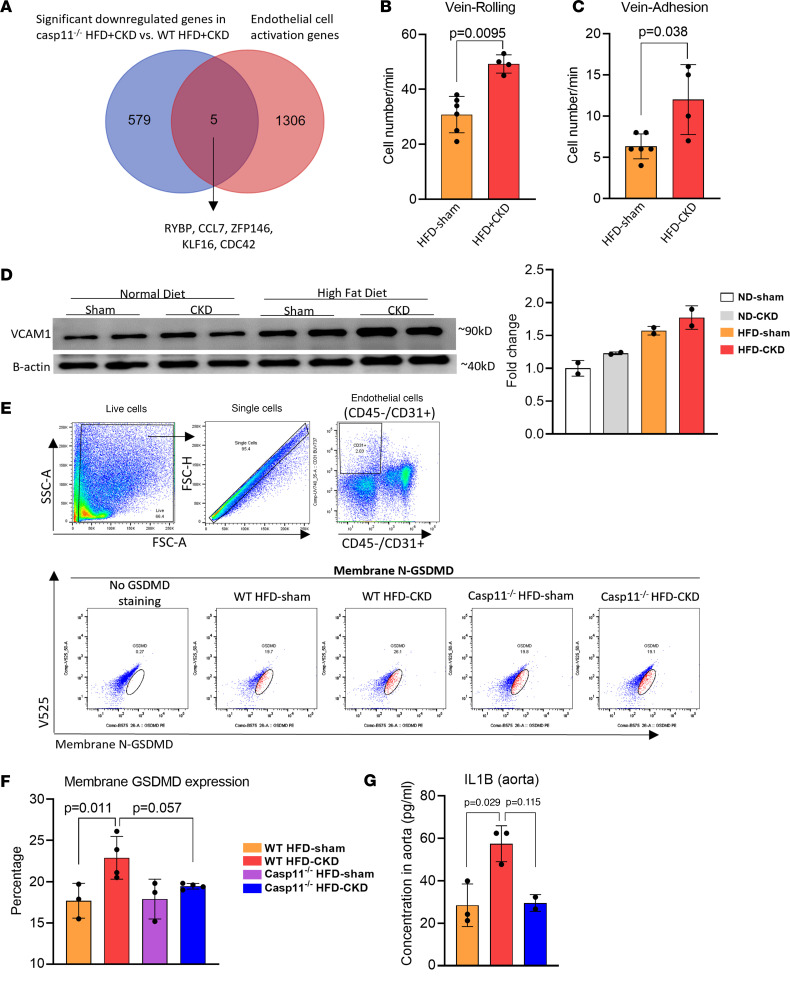
Caspase-11 deficiency decreases the cleavage of N-GSDMD in the aortas of HFD+CKD mice. (**A**) Five endothelial cell activation genes were identified in Venn diagram of 584 significantly downregulated genes in *casp11*^–/–^ aortas and 1,311 endothelial cell activation genes identified in the literature (*P* < 0.05, log_2_FC < –1). (**B** and **C**) Intravital microscopy was used to examine peripheral blood cell rolling and adhesion in the cremaster muscle vein in male mice (*n* = 4–6). (**D**) Western blot analysis showed that HF+CKD increased the expression of endothelial cell adhesion molecule *VCAM-1* in aortas compared with CKD and HFD-sham controls, suggesting that HFD+CKD activates aortic endothelial cells. (**E**) Flow cytometry gating analysis was used on mouse aorta cells to examine membrane GSDMD expression (*n* = 3–4). (**F**) Quantification of N-GSDMD expression was performed in endothelial cells (CD45^–^CD31^+^) in WT and *casp11*^–/–^ HFD+CKD and HFD-Sham mouse aortas. (**G**) IL-1β secretion in the aorta of WT and *casp11*^–/–^ HFD+CKD and HFD-Sham mice were quantified by ELISA. The Mann Whitney *U* test was used in **B** and **C**. The Kruskal-Wallis test with Benjamini and Hochberg multiple-comparison method was used to control the overall FDR of 5% (**F** and **G**).

We hypothesized that increased recruitment of monocytes and macrophages into the aorta in CKD+HFD WT mice results from CKD+HFD-induced EC activation. Our recent paper identifies a set of EC activation genes collected from 28 EC transcriptomic data sets activated by various DAMPs (48). Figure 5A shows that *CASP11*^–/–^ downregulated 5 EC activation genes, suggesting the role of *CASP11* in promoting EC activation. To verify this finding, we used an intravital microscope as an in vivo EC activation model (49) to examine blood leukocyte adhesion to the endothelium of cremaster muscle veins in male mice. We found that HFD+CKD significantly increased blood leukocyte rolling and adhesion in male mouse cremaster vessels compared with WT HFD-sham, suggesting that HFD+CKD activates ECs (Figure 5, B and C). Additionally, HFD+CKD further increased *VCAM-1* expression compared with ND-sham and ND-CKD, respectively (Figure 5D). These results suggest that HFD+CKD activates aortic ECs and upregulates the expression of EC adhesion molecules, which contribute to increased recruitment of monocytes and macrophages into the aorta.

Furthermore, we used flow cytometry to analyze N-terminal GSDMD (N-GSDMD) expression on CD45^–^CD31^+^ aortic ECs. Of note, *GSDMD* is a cytosolic protein. N-*GSDMD* can be cleaved by *CASP1* and *CASP4/11*. The N-*GSDMD*, but not the cytosolic full-length *GSDMD*, is expressed on the plasma membrane (50). HFD+CKD increased N-*GSDMD* expression, and *CASP11*^–/–^ decreased HFD+CKD-induced N-*GSDMD* expression on aortic CD45^–^CD31^+^ ECs (Figure 5, E and F). It has been reported that *CASP4/11* has a function to activate *NLRP3*-*CASP1*–mediated processing and secretion of *IL1B* via *GSDMD*, suggesting that *CASP4/11* acts as an upstream regulator for *NLRP3* inflammasome and *CASP1* activation (51), and that activated *CASP4/11* is less efficient in directly processing pre-*IL1B* into mature *IL1B* (52). Indeed, ELISA results show that *IL1B* secretion in the aorta was decreased by *CASP11* deficiency (Figure 5G). These results suggest that, with ECs as an innate immune cell model, *CASP4/11* deficiency makes less *IL1B* secretion via the N-GSDMD protein pore on the plasma membrane.

### LPS transfection into human aortic ECs (HAECs) results in Casp4 activation and increases N-GSDMD expression on the plasma membrane.

**Figure 6:**
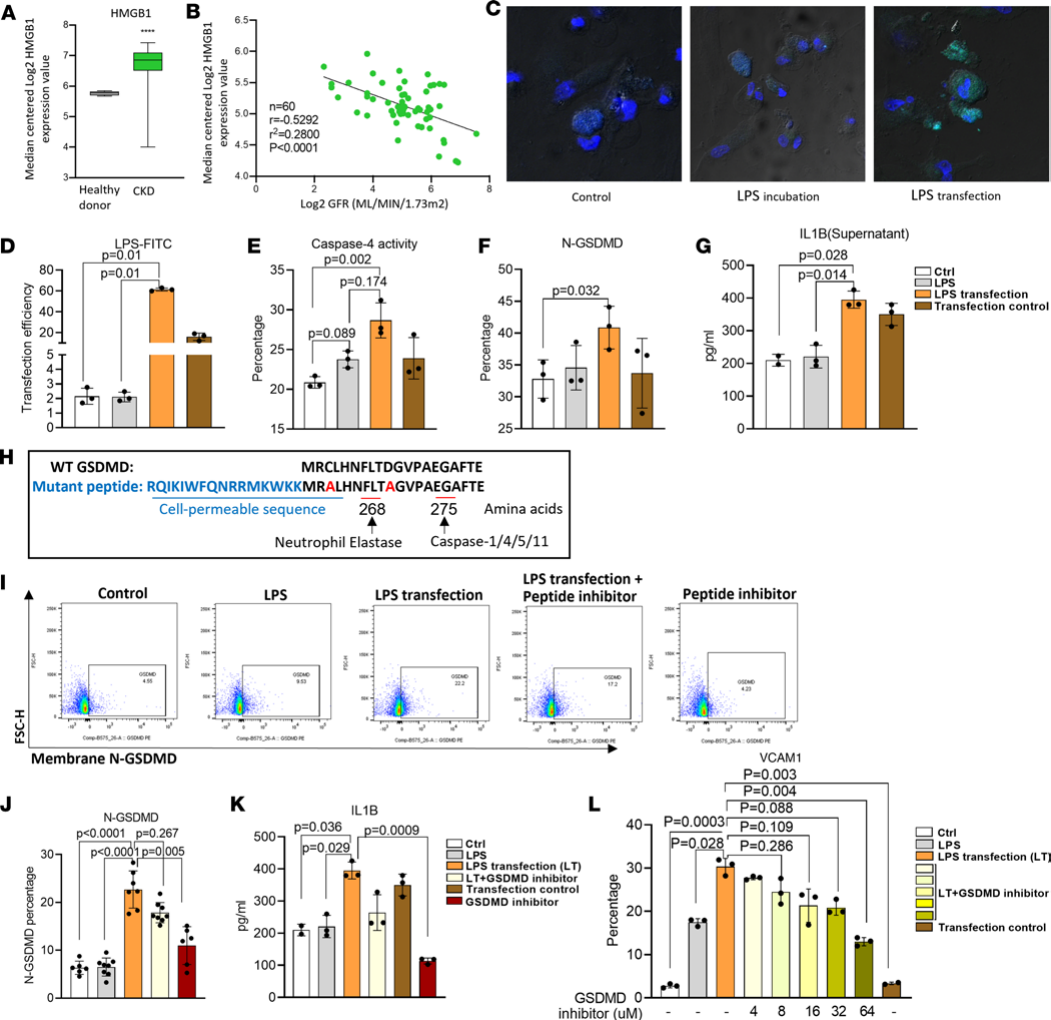
Cytosolic LPS is increased in the aorta of HFD+CKD and activates the casp4/11-GSDMD pathway. (**A**) The expression of LPS endocytic protein HMGB1 was increased in the kidneys of patients with CKD. (**B**) The expression of HMGB1 was negatively correlated with GFR (GSE9493, GSE66494). (**C** and **D**) The LPS-FITC (2 μg/mL) was transfected into human aortic endothelial cell (HAECs) using FuGENE for 16 hours. The transfection efficiency was detected by flow cytometry (**D**) and verified by visualization with confocal microscopy images (**C**) (40× magnification). (**E** and **F**) HAECs were treated with blank control, direct LPS stimulation (2 μg/mL), LPS transfection (2 μg/mL), and transfection control. The activity of casp4 (**E**) and cleavage of N-GSDMD (**F**) were examined by FLICA and flow cytometry. (**G**) The secretion of IL-1β in the supernatant was determined by ELISA. (**H**) The design of a new competitive inhibitor of GSDMD cleavage. (**I** and **J**) The inhibition efficiency detection of GSDMD peptide inhibitor (4 μM) was examined by flow cytometry (*n* = 6). (**K**) The secretion of IL-1β via the N-GSDMD protein channel into the supernatant of LPS-transfected HAECs was detected in the presence and absence of GSDMD cleavage inhibitor by ELISA (*n* = 3). (**L**) The expression of adhesion molecule *VCAM-1* was measured by flow cytometry in the LPD, LPS transfection, and LPS transfection after the addition of different concentrations of GSDMD inhibitor. Two-tailed Student’s *t* test was used in **A**. Pearson correlations were used in **B**. The Kruskal-Wallis test with Benjamini and Hochberg multiple-comparison method was used to control the overall FDR of 5% (**D**–**G** and **J**–**L**).

We hypothesized that HFD+CKD enhanced LPS endocytosis and that cytosolic LPS activates *CASP4/11*, which cleaves N-*GSDMD* and increases its membrane expression. We first found that extracellular LPS binding protein *HMGB1* was significantly increased in the kidneys of patients with CKD compared with healthy controls (Figure 6A), and higher expression levels of *HMGB1* were inversely correlated with GFR (Figure 6B), suggesting that *HMGB1*-mediated LPS endocytosis is associated with CKD progression, presumably via increasing *CASP4/11* activation. To study the endocytic LPS activation of *CASP4* in HAECs, we adopted a fluorescein isothiocyanate–conjugated (FITC-conjugated) LPS transfection experimental model (53) and verified it by flow cytometry and fluorescence microscopy (Figure 6, C and D). Cytosolic LPS increased *CASP4* activation detected by *CASP4* FLICA (Figure 6E), promoted N-*GSDMD* expression on the EC membrane (Figure 6F), and increased *IL1B* secretion into the supernatant of cultured HAEC (Figure 6G).

The *GSDMD* region from amino acid (aa) 268 to aa 275 is a key position for cleaving *GSDMD* and forming the N-*GSDMD* protein pore on the plasma membrane. We designed a cell membrane–permeable dominant negative peptide inhibitor by including 2 parts (54): (a) a 16 aa cell membrane permeable sequence as reported previously (55); and (b) C to A mutation at aa 268 and a D to A mutation at aa 275 for blocking *GSDMD* cleavage, presumably by *CASP4/11*, and *CASP8* (56), *CASP1* (cleaving *GSDMD* at aa 275) and neutrophil elastase (cleaving *GSDMD* at aa 268) (Figure 6H). *GSDMD* cleavage peptide inhibitor showed the decreased trend of LPS transfection–/*CASP4* activation–induced N-*GSDMD* expression on the EC membrane (Figure 6, I and J) and *IL1B* secretion into the supernatants of cultured HAECs (Figure 6K). Our data show that *GSDMD* peptide inhibitor not only suppresses N-GSDMD expression on the cell membrane but also inhibits *VCAM-1* upregulation induced by LPS transfection (Figure 6L).

### Palmitic acid in combination with UT indoxyl sulfate and LPS transfection upregulates VCAM-1 expression.

**Figure 7:**
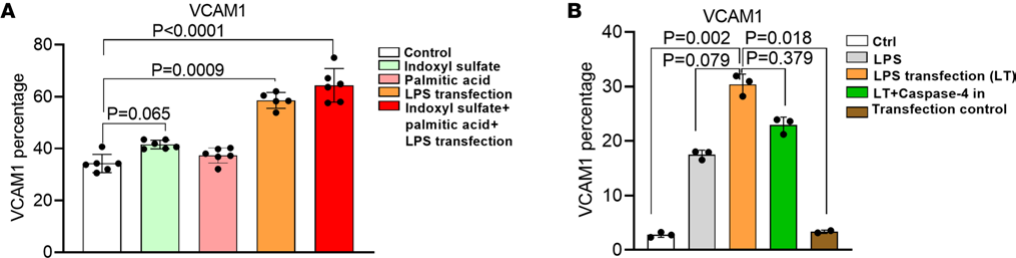
Cytosolic LPS-activated caspase-4 promotes human aortic endothelial cell activation via the caspase-4/GSDMD pathway. (**A**) HAECs were treated with CKD-related gut microbiota generated uremic toxin indoxyl sulfate (250 μM), palmitic acid (250 μM), and LPS transfection (2 μg/mL) for 4 hours. The expression of *VCAM-1* was examined by flow cytometry (*n* = 3). (**B**) The expression of adhesion molecule *VCAM-1* was measured by flow cytometry in the presence or absence of a casp4 inhibitor (50 μM). The Kruskal-Wallis test with Benjamini and Hochberg multiple-comparison method was used to control the overall FDR of 5% (**A** and **B**).

It has been reported that hyperlipidemia increases lipotoxic cholesterol and saturated fatty acids (57); the levels of all fatty acids, especially palmitic acid (PA), are increased in the plasma of patients with CKD (58); and saturated fatty acids, including PA, undergo intracellular crystallization and activate *NLRP3* (59). We adopted indoxyl sulfate as an in vitro CKD UT stimulation model for ECs (60). As shown in Figure 7A, indoxyl sulfate alone induced *VCAM-1* upregulation in HAECs. PA in combination with indoxyl sulfate and LPS transfection increased *VCAM-1* expression in HAECs. The results suggest the possibility that *CASP4* activation and N-*GSDMD* membrane expression can be separated from *VCAM-1* upregulation. LPS transfection–induced *VCAM-1* activation showed the decreased trend by *CASP4* inhibitor (Figure 7B). Taken together, our data suggest that cytosolic LPS-induced *CASP4* activation in HAECs mainly induces HAEC activation as judging by *VCAM-1* upregulation, which may be realized via *CASP4-GSDMD* secretome including *IL1B* signaling–mediated *VCAM-1* upregulation (61).

### LPS transfection increases mitoROS, and mitoROS inhibitor mitoTEMPO inhibits Casp4 activation, N-GSDMD expression on the plasma membrane, and VCAM-1 upregulation in HAECs.

**Figure 8:**
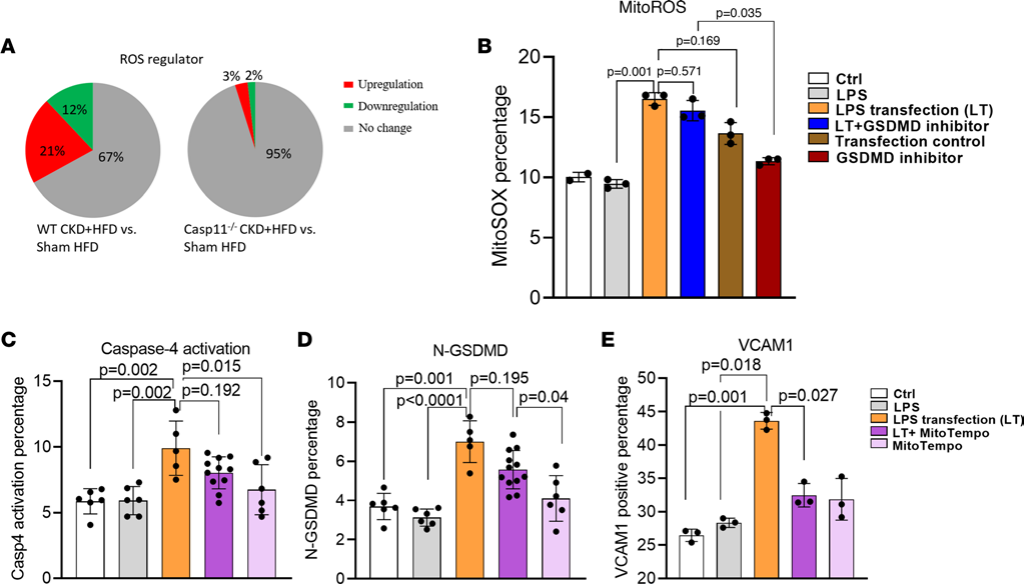
Mitochondrial ROS (mitoROS) generation is increased by cytosolic LPS, mitoROS promotes the caspase-4/GSDMD pathway, and casp4/GSDMD also promotes mitoROS generation. (**A**) In total, 165 ROS regulator genes from GSEA were screened in WT aorta RNA-Seq data and *casp11*^–/–^ CKD+HFD aorta RNA-Seq data. (**B**) The mitoROS level was detected using mitoSOX (5 μM) in LPS-transfected HAECs in the presence or absence of a GSDMD cleavage inhibitor (8 μM). (**C**–**E**) Casp4 activity (**C**). The expression levels of N-GSDMD (**D**) and adhesion molecule *VCAM-1* (**E**) were detected in LPS-transfected HAECs in the presence or absence of mitoROS inhibitor mitoTempo (1 μM) using flow cytometry. The Kruskal-Wallis test with Benjamini and Hochberg multiple-comparison method was used to control the overall FDR of 5% (**B**–**E**).

We found that WT HFD+CKD upregulated 21% of 165 ROS regulator genes; however, HFD+CKD *CASP11*^–/–^ reduced the upregulation of ROS regulator genes to 3% (Figure 8A), suggesting that *CASP11* plays significant roles in promoting the expression of ROS regulator genes in the HFD+CKD aorta. Additionally, oxidative stress pathways and mitochondrial complex expressions were modified in the HFD+CKD aorta (Supplemental Figure 3). Given that PA levels are increased in patients with CKD, and considering previous reports indicating that high doses and prolonged exposure to PA can increase mitochondrial ROS production and mitochondrial mass (62). Then, we hypothesized that mitochondrial ROS plays a positive feedback role in promoting *CASP4* activation (63). LPS transfection–induced mitoROS was relatively suppressed by a *GSDMD* peptide inhibitor (Figure 8B). In addition, mitoROS inhibitor mitoTEMPO showed a trend of inhibition of LPS transfection–induced *CASP4* activation (Figure 8C), N-GSDMD expression on the plasma membrane (Figure 8D), and *VCAM-1* upregulation (Figure 8E). Taken together, our results demonstrate that HFD+CKD upregulates ROS regulator gene expressions in the aorta via a *CASP4/11*-dependent manner and that LPS transfection induced mitoROS, inhibition of mitoROS decreased *CASP4* activation, N-*GSDMD* membrane expression, and *VCAM-1* upregulation, suggesting that mitoROS are functional upstream of *CASP4/11* activation.

### IL1B serves as the second-tier mechanism of HFD+CKD-promoted TI, promotes TI gene expression, and enhances Casp4–induced VCAM-1 expression and IL1B secretion in HAECs.

**Figure 9:**
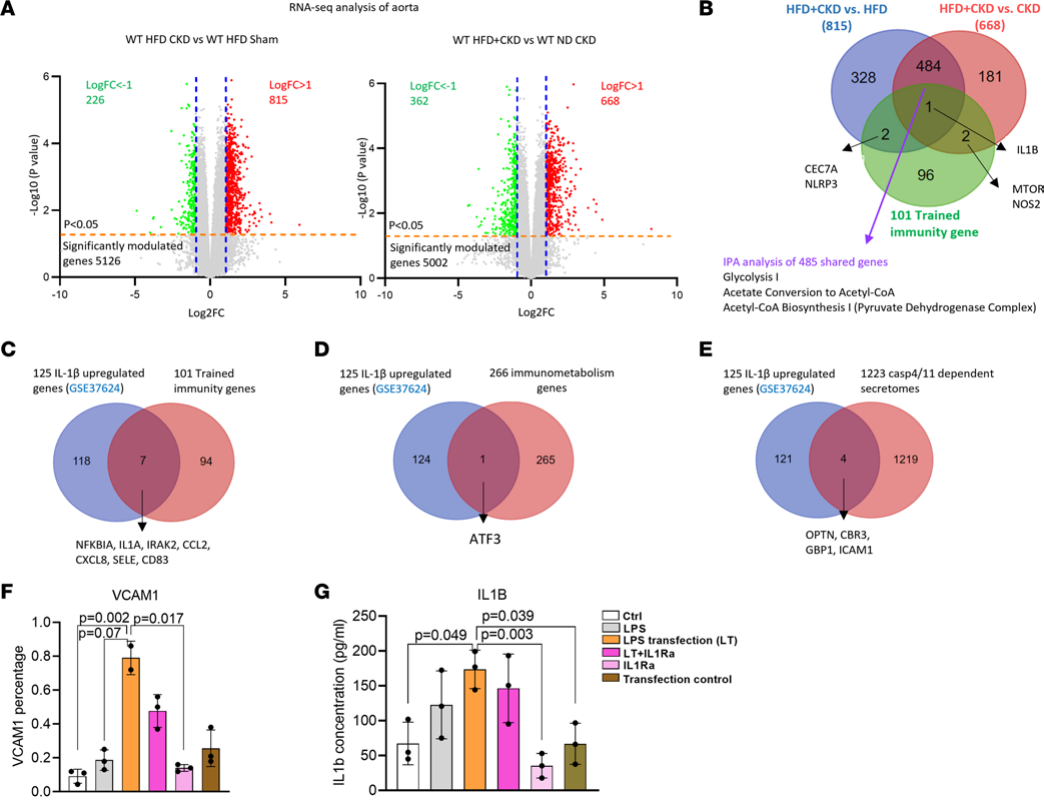
IL-1β released via caspase-4/N-GSDMD protein pores serves as the second step of HFD+CKD-promoted trained immunity, promotes trained immunity gene expression, and enhances caspase-4/11–induced *VCAM-1* expression and IL-1β secretion in HAECs. (**A**) Volcano plot analysis of bulk RNA-Seq data shows the differentially expressed genes in HFD+CKD versus HF and HFD+CKD versus CKD. (**B**) Venn diagram of upregulated genes in HFD+CKD versus HFD and HFD+CKD versus CKD and 101 trained immunity genes. (**C**–**E**) Venn diagram showed the common shared genes between 125 IL-1β–upregulated genes and 101 trained immunity genes (**C**), 266 immunometabolism genes (**D**), and 1,223 casp4/11-dependent secrotomes (**E**). (**F** and **G**) *VCAM-1* expression (**F**) and IL-1β secretion (**G**) were examined in LPS-transfected HAECs in the presence and absence of IL-1Ra (10 μM) (*n* = 3). Each experiment was repeated 3 times. The Kruskal-Wallis test with Benjamini and Hochberg multiple-comparison method was used to control the overall FDR of 5% (**F** and **G**).

To determine whether casp11 activation is associated with gene expression changes in the aortas of HFD+CKD mice, we performed RNA-Seq analysis. HFD+CKD upregulated 815 of 15,462 genes and 668 of 15,456 genes compared with HFD-sham and ND-CKD, respectively (log_2_FC > 1, *P* < 0.05) (Figure 9A). Then, we used the Venn diagram analysis and found that 485 genes were shared between HFD+CKD and HFD and between HFD+CKD and CKD; therefore, HFD+CKD upregulated 998 genes in total (815 + 668 –485 = 998) (Figure 9B). It has been reported that *HMGB1*/ *RAGE* mediates LPS endocytosis by binding to extracellular LPS (64) and that GBP*s* promote *CASP4/11*-dependent pyroptosis in response to cytosolic LPS (65). Therefore, we examined the upregulation of 7 *GBP* family members, *HMGB1*, and *RAGE* (Supplemental Figure 4A) and found that *GBP3* and *GBP6* were upregulated in HFD+CKD versus CKD and HFD+CKD versus CKD, respectively, which may contribute to HFD+CKD-promoted LPS endocytosis and cytosolic LPS activation of *CASP4/11*. However, we did not find upregulation of *HMGB1* (slightly decreased) or *RAGE* in HFD+CKD versus HFD, nor in HFD+CKD versus CKD, suggesting that *HMGB1/RAGE* may facilitate LPS endocytosis without significant upregulation. The single-cell RNA-Seq (single-cell portal at the Broad Institute of MIT and Harvard, Cambridge, Massachusetts, USA; https://singlecell.broadinstitute.org/single_cell) in the aortas of HFD fed mice showed upregulated LPS endocytic machinery components in ECs (Supplemental Figure 4B).

As we previously reported, the upregulation of caspases and inflammasome pathway genes is the first mechanism for activating *CASP1* and *CASP4/11* pathways (66). Therefore, we examined the transcriptomic changes of canonical and noncanonical inflammasome genes. Our data show that HFD+CKD versus HFD upregulated 6 canonical inflammasome genes, including *IL1B*, *ITPR2*, *CYBB*, *NAMPT*, *MAVS*, and *TXN2*, and 3 noncanonical inflammasome genes, including *IL1B*, *SCL25A22*, and *GBP6*. Similarly, HFD+CKD versus CKD upregulated 6 canonical inflammasome genes, including *IL1B*, *ITPR2*, *MAVS*, *NAMPT*, *OAS2*, and *TXN2*, and 4 noncanonical inflammasome genes, including *IL1B*, *SLC25A22*, *GBP6*, and *GBP3* (Supplemental Figure 4C).

Hyperlipidemia plays significant roles in promoting TI, and Western diet triggers canonical inflammasome NLRP3-dependent innate immune reprogramming (67). Therefore, we examined the transcriptomic changes of 101 TI-related genes from the most updated TI database (68). The Venn diagram shows that HFD+CKD versus HFD upregulated 3 TI genes, including *IL1B*, *CLEC7A*, and *NLRP3*. Also, HFD+CKD versus CKD upregulated 3 TI genes, including *IL1B*, *mTOR*, and *NOS2* (Figure 9B). Furthermore, the ingenuity pathway analysis (IPA) shows that HFD+CKD versus HFD and HFD+CKD versus CKD upregulated 12 TI pathways, including the superpathway of cholesterol biosynthesis, glycolysis I, superpathway geranylgeranyldiphosphate biosynthesis I (via mevalonate), cholesterol biosynthesis III (via desmosterol), cholesterol biosynthesis II (via 24, 25 dihydrolanosterol), cholesterol biosynthesis I, pentose phosphate pathway, mevalonate pathway I and signaling pathway, acetyl-CoA biosynthesis I (pyruvate dehydrogenase complex), acetate conversion to acetyl-CoA, and NAD phosphorylation and dephosphorylation (Supplemental Figure 5). These results suggest that HFD+CKD promotes TI in the mouse aorta.

It has been reported that *IL1B* not only serves as a TI readout cytokine (69) but also promotes TI (69, 70). We hypothesized that *IL1B* released from the *GSDMD* protein pore on the plasma membrane can further enhance TI and amplify inflammation in HAECs. In Figure 5G, HFD+CKD-induced *IL1B* in the aorta was decreased in *CASP11*^–/–^ mice. Then we determined how many of 101 TI-related genes were upregulated by *IL1B* stimulation in human ECs. As shown in the Venn diagram (Figure 9C), 7 of 125 *IL1B* upregulated genes in the human ECs (GSE37624) data set were TI-related genes, including *NFKBIA*, *IL1A*, *IRAK2*, *CCL2*, *CXCL8*, *SELE*, and *CD83*. In addition, we also analyzed expressions of 266 immunometabolism regulator genes in human *IL1B*-stimulated ECs (71) and found that immunometabolism transcription factor *ATF3* was upregulated (Figure 9D), which was shown to interact with *RGS7* and histone acetyltransferase Tip60 and form a unique pathway to promote hepatic steatosis (72), suggesting that *IL1B* promotes immunometabolism reprogramming, histone acetylation, and gene expression. Moreover, we also analyzed expressions of 1,223 *CASP4-GSDMD* secretome genes in human *IL1B*–stimulated ECs and found that OPTN (promotes type I IFN generation and *IFNA/B* receptor signaling; ref. 73), *CBR3*, *GBP1* (a cytosolic LPS-binding protein to promote *CASP4/11* activation; ref. 74), and *ICAM1* (an EC activation adhesion molecule and inflammation promoting gene; ref. 75) were upregulated (Figure 9E). Then, we hypothesized that *IL1B* secreted from the N-GSDMD protein pore could stimulate HAECs again. We found that the *IL1B* receptor antagonist IL-1RA showed a trend of inhibition of LPS transfection–induced *VCAM-1* expression and IL-1β secretion (Figure 9, F and G) in HAECs. Taken together, our results demonstrate that HFD+CKD upregulate the expression of 998 genes with upregulation of 5 TI genes, including *IL1B*, *NLRP3*, *CLEC7A*, *mTOR*, and *NOS2*, in the aorta; *IL1B* not only serves as a cytokine readout for *CASP4/11* activation and the N-GSDMD protein pore-secretome but also acts as the second step for HFD+CKD-promoted TI to accelerate vascular inflammation.

## Discussion

We proposed the concepts that UTs are DAPMs (13). CKD-UTs promote aortic vascular inflammation via enhancing various secretomes (15). *CASP1* senses UTs in CKD and promotes neointima hyperplasia (14) and vascular smooth muscle cell phenotyping switch (76). Hyperlipidemia acts synergistically with other disease risk factors in establishing TI and hyperactivation of inflammation (67). Regardless of the significant progress, several important considerations remain poorly addressed: first, how hyperlipidemia and CKD achieve synergy in accelerating vascular inflammation; second, how intracellular gram-negative bacterial infections in CKD and elevated LPS levels derived from gut gram-negative bacteria promote intracellular inflammatory mechanisms; and third, whether TI has coupled mechanisms from the first step (*CASP4/11* activation) to the second step (promotion by *IL1B* and other *CASP4/11-GSDMD* secretome) to be amplified. To address those considerations, we performed aortic pathological analysis and RNA-Seq in the *CASP11*^–/–^ mice. We found that HFD+CKD increases plasma LDL-VLDL, aortic cytosolic LPS levels, *CASP11* activation, and TI pathways in the aorta. In contrast, *CASP11*^–/–^ decreases aortic neointima hyperplasia, recruitment of monocytes and macrophages into the aorta, and secretion of *CASP11-GSDMD*–mediated secretome cytokines (*CCL22* and *PCSK9*) in the plasma. *CASP11*^–/–^ further decreases N-GSDMD membrane expression on mouse aortic CD45^–^CD31^+^ ECs and reduces mouse aortic *IL1B* levels. To mimic the increased cytosolic LPS level in vivo, we did LPS transfection into HAECs, which results in *CASP4* activation, N-GSDMD membrane expression, and mitoROS generation. MitoROS inhibitor mitoTEMPO inhibits *CASP4/11* activation, N-GSDMD membrane expression, and *VCAM-1* upregulation in HAECs. Importantly, *IL1B* serves as the second step, and positive feedback of HFD+CKD-promoted TI, promotes TI gene expression, and enhances *CASP4*-induced *VCAM-1* expression and *IL1B* secretion in HAECs. Based on current understanding, although canonical and noncanonical inflammasome pathways share similar downstream signaling, including *GSDMD* and *IL1B*, the *Casp11*–induced noncanonical inflammasome pathway may be located upstream of canonical inflammation (77, 78). Our data demonstrate that *Casp11* promotes TI in conditions of HFD and CKD. However, it needs to be further determined whether the *Casp11*–promoted TI is a canonical inflammasome dependent or independent phenotype.

**Figure 10:**
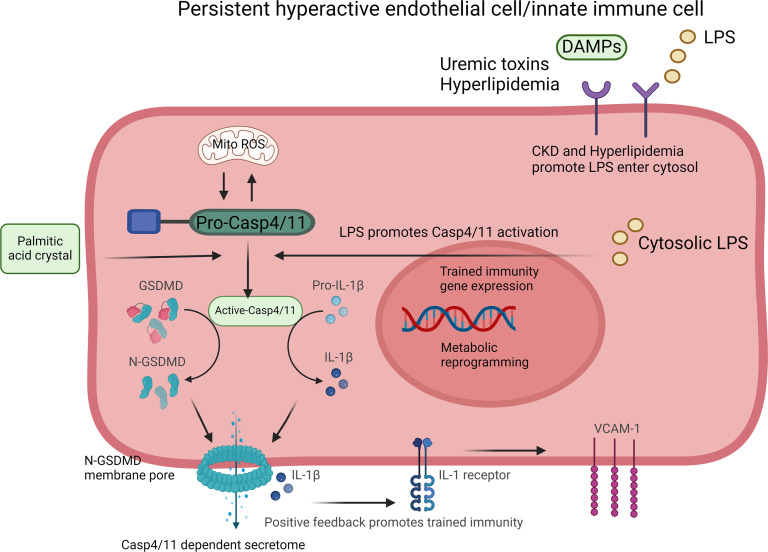
Our working model. HFD+CKD (UTs) promote extracellular LPS enter aortic cell cytosol, increase intracellular gram-negative bacterial infections in CKD, increase intracellular crystallization of CKD-elevated palmitic acid, activate casp4/11 and N-GSDMD membrane expression, increase secretion of IL-1β and other casp11-GSDMD secretome, and upregulate TI genes in aortic cells; after sensing intracellular LPS, palmitic acid stimulation, and UT indoxyl sulfate stimulation, casp11 gets activated and cleaves N-GSDMD and promotes N-GSDMD membrane expression in aortic endothelial cells.

We proposed a working model (Figure 10) to integrate all the results. First, our data show that HFD+CKD (UTs) promote extracellular LPS enter aortic cell cytosol, increase intracellular gram-negative bacterial infections in CKD (79, 80), increase intracellular crystallization of CKD-elevated PA, activate *CASP4/11* and N-*GSDMD* membrane expression, increase secretion of *IL1B* and other *CASP11-GSDMD* secretome, and upregulate TI genes in aortic cells. Second, *CASP11* deficiency decreases HFD+CKD-induced upregulations of immunometabolic genes and TI genes and secretion of *IL1B* and other *CASP11-GSDMD* secretome, suggesting that *CASP11* promotion of HFD+CKD induced TI. Third, after sensing intracellular LPS, PA stimulation, and UT indoxyl sulfate stimulation, *CASP11* gets activated and cleaves N-*GSDMD* and promotes N-*GSDMD* membrane expression in aortic ECs. These results have once again demonstrated our concept that ECs are innate immune cells (9). Fourth, our data have demonstrated for the first time to our knowledge that *IL1B* serves as the second-step TI mechanism to amplify HFD+CKD-accelerated EC activation and vascular inflammation.

*GSDMD* peptide inhibitor suppresses *VCAM-1* upregulation induced by LPS transfection, suggesting that N-*GDSMD* membrane expression has a function in promoting *VCAM-1* upregulation, presumably via secretion of *IL1B* and other *CASP4/11-GSDMD* secretomes. Our data have further verified that *IL1B* receptor antagonist inhibit *VCAM-1* upregulation. Therefore, *IL1B* has 3 roles: (a) cytokine readout for *CASP4/11-GSDMD* pathway activation; (b) TI readout; and (c) second-step TI promoter for HFD+CKD-accelerated vascular inflammation. Of note, due to the high incidence of CKD in females (81), we acknowledge that only using male mice in this study is a major limitation and that the phenotype of females regarding HFD+CKD-promoted TI should be further studied. Taken together, our results provide insights into the roles of gram-negative bacteria in gut-generated LPS and intracellular gram-negative bacterial infections in CKD and hyperlipidemia pathologies activating *CASP4/11-GSDMD* and *IL1B* pathways. This activation leads to the acceleration of vascular inflammation via enhanced TI in 2 tiers, highlighting therapeutic targets for the future development of treatments for CVDs, inflammation, immune diseases, transplantation, aging, and cancers.

## Methods

### Sex as a biological variable.

Our study exclusively examined male mice. It is unknown whether the findings are totally relevant for female mice.

### Reagents and antibodies.

Casp4 inhibitor Z-LEVD-FMK was bought from Biovision (catalog 1144). MitoTEMPO (Enzo, ALX-430-150) was dissolved in DMSO and used at a final concentration of 1 μM. IL-1R antagonist (Cayman, 21349) was used at final concentration of 10 μM. Western blot antibodies included: anti-CD54/ICAM-1 antibody (Cell Signaling Technology, 49155), anti-VCAM-1 (Abcam, ab134047), anti-GSDMD (Novus, NBP2-33422), anti–N-GSDMD (Santa Cruz Biotechnology Inc., sc-393581), mouse IL-1β/IL-1F2 (R&D, AF401), and anti-casp1 (Santa Cruz Biotechnology Inc., sc-392736). Flow cytometry antibodies were bought from BD Biosciences except for anti–GSDMD-PE (Santa Cruz, H11), anti–VCAM-1 PECy7 (BioLegend, 105720), anti-CD11b BUV395 (BD Biosciences, 563553), anti-CD45 APC Cy7 (BioLegend, catalog 103116), anti-CD31 BUV737 (BD Biosciences, 612802), and anti-F4/80 Alexa Fluor 647 (BD Pharmingen, catalog 565853).

### Animal care.

The *casp11*^–/–^ male mice were in a C57BL/6 background and were purchased from the Jackson Laboratory (stock no. 024698). Both WT and *casp11*^–/–^ male mice were weaned at 3 weeks of age and carried out surgery and a HFD at week 9. At week 18, mice were sacrificed and tissues were collected for analysis.

### Hyperlipidemia-5/6 nephrectomy CKD model.

The 2-step renal ablation procedure was used to create the CKD model as described before (14). At week 9, the left kidney was exposed, and 80%–90% of the kidney cortex was ablated, except the renal artery, vein, and pelvis. After 1 week, a right kidney nephrectomy was performed. Sham control animals received sham operations without renal injury. At week 18, the mice were sacrificed, and samples collected for further analysis. In addition, mice were maintained on a ND (5% fat, Labdiet 5001) or a HFD (0.2%[w/w] cholesterol, and 20% [w/w] fat, Test Diet AIN-76A) from week 8 to week 18.

### Carotid artery ligation.

Two weeks after right nephrectomy, the left common carotid artery (LCA) was partially ligated to create neointima hyperplasia, as described previously (82). Briefly, a 5 mm vertical incision was made in the middle of the neck to bluntly dissect the bifurcation and expose the 4 distal branches of the LCC artery under a microscope. Three of 4 caudal branches of LCA were ligated with a 6-0 silk suture. The carotid artery ligation was carried out in both CKD and sham mice.

### BUN detection.

The BUN was detected by using the Stanbio Urea Nitrogen kit (STANBIO, 0580). The creatinine was detected using the Serum Creatinine Detection kit (Arbor Assays, KB02-H1). Briefly, the blood of mice was collected at the weeks 11 and 18. After collection, the blood was centrifuged at 13,000*g* at 4°C, and the upper layer, which is plasma, was collected for BUN and creatinine examination following the manufacturer instructions.

### Mouse genotype.

Mouse genotype was confirmed with end-point PCR on genomic DNA from mouse toes by using Extracta DNA Prep for PCR (Quanta, 95091). Briefly, mouse toes were digested in 50 μL extraction reagent at 98^°^C for 30 minutes, after which 50 μL stabilization buffer was added. DNA was used to perform PCR or stored at 4°C. Then, 2% agarose gel was imaged by the Foto analyst image system. The following primer sequences were used for the *casp11*^–/–^ mice: mutant reverse sequence: CGC TTC CTC GTG CTT TAC GGT AT; common sequences: ACA ATT GCC ACT GTC CAG GT; and WT reverse sequences: CAT TGC TGA CCT TAT TTC TGT ATG G. The PCR running protocol was 94°C for 2 minutes and then 10 cycles of the following: 94°C for 20 seconds, 65°C for 15 seconds, and 68°C for 10 seconds before 28 cycles of 94°C for 15 seconds and 60°C for 15 seconds. The DNA sizes of mutant casp11 is 650 bp and WT casp11 is 495 bp.

### Aortic tissue single-cell suspension.

Aortas were collected after perfusion and stored in DMEM-low medium (GE Life Sciences) supplemented with 20% FBS until digestion. Then aortas were rinsed with phosphate buffer solution (PBS) and dissected in enzymatic cocktails containing FBS, N-2-hydroxyethylpiperazine-N-2-ethane sulfonic acid (HEPES, Thermo Fisher Scientific), hyaluronidase type 1-S (MilliporeSigma), collagenase types I and XI (MilliporeSigma) at 37°C for 30 minutes. After which, the suspension was filtered with 70 μM filters, washed twice, and resuspended in HBSS supplemented with 2% FBS. Then the samples were ready for flow cytometry analysis.

### Flow cytometry.

HAECs were collected by trypsin, washed, and resuspended in FACS buffer (2% FBS in HBSS). The HAECs and aortic single-cell suspension were fixed or not with 1% paraformaldehyde (PFA) at 4°C and stained with surface markers for 15 minutes at 4°C. Cells were washed twice and stained with intracellular enzymes using *Casp4* FLICA (ImmunoChemistry, 913) for 1.5 hours at 37°C. Data were collected using a BD LSRII flow cytometer and DIVA software (BD Biosciences) and analyzed using FlowJo (BD Biosciences).

### Verhoeff–van Gieson staining of the carotid artery.

The carotid artery was perfused, collected, cleaned from fat and connective tissue, and fixed with 4% PFA overnight. The samples were transferred to 70% ethanol and rinsed in PBS. Samples were sent to the AML laboratory for paraffin embedding, and the cross-sections were cut into 5 μM thickness; Van Gieson’s staining was performed. The carotid samples were imaged under a microscope.

### Intravital microscopy.

Intravital microscopy was used to determine leukocyte rolling and adhesion in vivo*,* as previously described (83). Briefly, male mice were anesthetized, and the cremaster muscle was exposed. Venules with diameters between 38 and 43 μM were used for rolling and adhesion measurement. The venules were visualized under a microscope (Olympus BX51WI) with a digital camera (Plympus DP80) by using CellSens Dimension software (Olympus). Adhesion cells were determined as the cells failed to pass the imaginary line within 1 minute, and the cells that rolled past the imaginary line were determined as rolling cells. Rolling and adhesion cells were measured in 3 venules, and the mean numbers are recorded.

### RNA-Seq analysis.

The whole aorta of mice was collected. The RNA extraction and RNA-Seq were performed by GENEWIZ. Libraries containing Illumina adapter with TruSeq HT indexes were subsequently pooled and loaded into the Hiseq 2500. Single-end reads at 75 bp with 30 million reads per sample were generated for bioinformatic analysis.

### Public data sets.

TI genes were collected from TIDB (https://academic.oup.com/database/article/doi/10.1093/database/baab041/6318070) (68) and screened them in our RNA-Seq data. The human IL-1β–stimulated EC data set was obtained from the NIH-GEO data set GSE37624. Renal patient data were collected from the Nephroseq database, a comprehensive database at the University of Michigan O’Brien Renal Center (Ann Arbor, MI, USA). For our analysis, we utilized the data set described in Ju et al. (84). NephroSeq conducted differential expression profiles using a 2-tailed Student’s *t* test for 2-class differential expression analyses and Pearson correlations for all genes in each data set against various clinical properties, such as GFR.

### IPA and metascape analysis.

IPA was used to characterize the clinical relevance and molecular and cellular functions related to the genes in our RNA-Seq data. Differentially expressed genes were collected and uploaded to IPA for further analysis. Gene lists were uploaded to the Metascape website: https://metascape.org/gp/index.html#/main/step1

### Cytokine array.

Mouse plasma was collected as described before. The samples from 3 mice in each group were pooled together, and we carried out the experiments following the manufacturer’s protocol (R&D, ARY028). Membranes were incubated with enhanced chemiluminescence (ECL) substrates (Thermo Fisher Scientific, 34578) and imaged by a Fujifilm LAS-4000. Protein expression levels were quantified using ImageJ software.

### Casp11 activity assay.

Protein from aortic tissue was collected as described above. In total, 50 μg of protein was used to detect *Casp4* activity using the *Casp4* activity assay (Abcam, ab65659) following the manufacturer’s instructions.

### ELISA.

After euthanizing mice, plasma and aortas were collected for further experiments. The aorta was ground into powder in a mortar while using liquid nitrogen to keep the sample chilled. Then, samples were collected in Eppendorf tubes, and 100 μL of radioimmunoprecipitation assay (RIPA) buffer (Abcam, ab156034) was added. Next, the sample was sonicated at 40 mA for 15 seconds and centrifuged for 2 minutes at 17,000*g* at 4°C. Protein concentration was quantified using the colorimetric Pierce Bicinchoninic Acid (BCA) protein assay kit (Thermo Fisher Scientific 23225). In total, 300 μg of protein was used to detect IL-1β using an IL-1β ELISA kit (R&D systems, MLB00C). In total, 100 μL of mouse plasma was used to detect IL-1β using the IL-1β ELISA kit. Cell culture supernatant was collected and used for IL-1β detection (R&D systems, DLB50). The cell culture medium was concentrated by using Amicon Ultra tubes (Merck Millipore, R0JB86182). In total, 100 μL of the centrifuged medium was used for IL-1β detection. The original IL-1β concentration in the medium was calculated. Aortic proteins (50 μg) were used to detect LPS levels using the LPS ELISA kit (MyBioSource, MBS700021) following the manufacturer’s instructions.

### HAEC culture.

HAECs (Lonza, CC2535) were cultured in medium M199 (Hyclone Laboratories) supplemented with 20% FBS (HyClone), EC growth supplement (ECGS, 50 μg/mL) (BD Biosciences), heparin (50 μg/mL), and 1% penicillin, streptomycin, and amphotericin (PSA). HAECs were grown in 0.2% gelatin-coated flasks, dishes, and plates. Passage 9 of HAECs was used for experimental analysis.

### LPS transfection and GSDMD siRNA transfection.

HAECs were cultured on plates or dishes in M199 medium supplemented with 10% FBS. HAECs were primed with Pam3C (2 μg/mL, InviviGen, vac-pms) for 6 hours in M199 medium supplemented with 2% FBS; then, HAECs were transfected with 2 μg/mL LPS (Invivogen, tlrl-3pelps) in the absence or presence of transfection reagent FuGENE (3 μL/mL, Promega, E2311) for 16 hours. After which, HAECs were used for further experiments. HAECs were transfected with 10, 25, 50 nM of control siRNA or GSDMD siRNA (Horizon) with DharmaFECT transfection reagent (2.5 μL/mL) for 72 hours (mRNA examination) and 96 hours (protein examination). Then, HAECs were used for further experiments.

### Mitochondrial ROS detection by using MitoSOX.

We used the mitoSOX to examine the mitoROS level as previously described (82). Briefly, HAECs were incubated with mitoSOX (5 μM) at 37°C for 10 minutes before treatment. After incubation, HAECs were washed with PBS twice and collected for mitoROS measurement using flow cytometry.

### Confocal microscopy.

Confocal microscopy was used to examine LPS transfection in HAECs. Briefly, passage 9 HAECs were split in 8-well ibidi plates. Then HAECs were transfected with 2 μg/mL LPS-FITC (MilliporeSigma, F3665) in the absence or presence of FuGENE (Promega, E2311) for 16 hours. Following that, HAECs were washed and fixed with 1% PFA. Then HAECs were mounted with NucBlue Live Cell Stain ReadyProbes Reagent (Invitrogen, 2008165) and visualized under confocal microscopy.

### RNA extraction and real-time PCR (RT-PCR).

RNA from powdered aortic tissue or HAECs was extracted with the miRNeasy Mini Kit (Qiagen, 217004) following the manufacturer’s instructions. Then the RNA concentration was measured by Nanodrop 2000 (Thermo Fisher Scientific). In total, 1 μg of RNA was used to synthesize the complementary DNA (cDNA) using the High-Capacity cDNA Reverse Transcription Kit (Applied Biosystems, 4368814). RT-PCR was performed with iTaq Universal SYBR Green Supermix (Bio-Rad). Samples were amplified by 40 cycles of 5 seconds at 95°C and 30 seconds at 60°C. Results were calculated using the ^ΔΔ^Ct method relative to the reference control gene of β-actin. The primer sequences are shown in Table 3.

### Protein extraction and Western blot.

Protein extracts were collected from HAECs and aortic tissue. Protein concentrations were determined with the BCA assay. Then proteins were separated on SDS-PAGE and transferred onto nitrocellulose membranes. Membranes were blocked with 5% nonfat milk in Tris-buffered saline containing 0.1% Tween 20 (TBST, 50 mM Tris [pH 7.5], 150 mM NaCl, and 0.1% Tween 20 [v/v]). Then the membranes were washed twice with TBST and incubated with primary antibodies overnight at 4°C. Following 3 washes with TBST, the membranes were incubated with horseradish peroxidase–labeled secondary antibodies for 1.5 hours at room temperature. Then, the membranes were incubated with ECL (Thermo Fisher Scientific, 34578), imaged with Fujifil LAS-4000. The protein density was quantified with ImageJ (NIH).

### Statistics.

Data were expressed as the mean ± SEM throughout the manuscript. Check the data normality by using GraphPad Prism 8. For nonparametric comparisons in sample size *n* < 6 between 2 groups, the Mann Whitney *U* test was used. For nonparametric comparisons across multiple groups, the Kruskal-Wallis test with Benjamini and Hochberg multiple-comparison method was used to control the overall FDR of 5%. The data shown were representative of 2–3 independent experiments, including analysis from RT-PCR, flow cytometry, and Western blot. *P* < 0.05 was considered statistically significant.

### Study approval.

All animal experiments were performed in accordance with the IACUC guidelines and were approved by the IACUC of Lewis Katz School of Medicine at Temple University with protocol no. 5006.

### Data availability statement.

The RNA-Seq data sets generated for this study have been deposited to NCBI with accession ID PRJNA1106364. Values for all data points shown in graphs can be found in the Supporting Data Values file.

## Author contributions

Y Sun and YL contributed equally and are co–first authors. Y Sun contributed from 2016 to 2021 and YL contributed from 2021 to 2024, which determined authorship order. Y Sun and YL carried out the data gathering and data analysis and prepared the tables and figures. FS, LL, Y Shao, KX, CD, RC, HS, XJ, HZ, and HW aided with analysis of the data. XY supervised the experimental design, data analysis, and manuscript writing. All authors read and approved the final manuscript.

## Supplementary Material

Supplemental data

Unedited blot and gel images

Supporting data values

## Figures and Tables

**Table 3:**
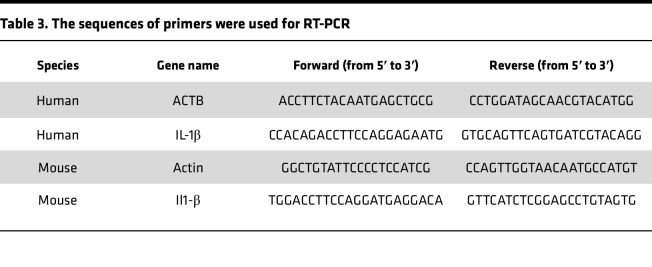
The sequences of primers were used for RT-PCR

**Table 1:**
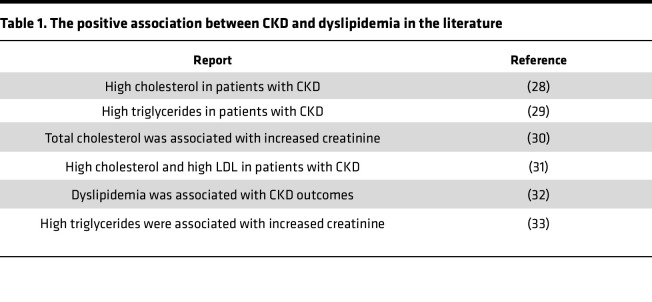
The positive association between CKD and dyslipidemia in the literature

**Table 2:**
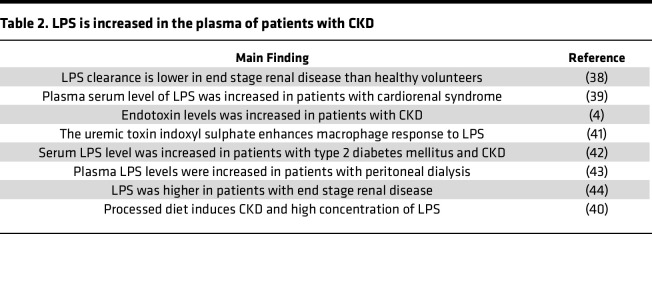
LPS is increased in the plasma of patients with CKD

## Version 1 07/18/2024

In-Press Preview

## Version 2 08/22/2024

Electronic publication
