# Supplementary material for: Caspase-4/11 promotes hyperlipidemia and chronic kidney disease–accelerated vascular inflammation by enhancing trained immunity
Source: JCI Insight. 2024 Jul 18;9(16):e177229. doi: 10.1172/jci.insight.177229 (PMC11343595; doi:10.1172/jci.insight.177229)
Supplement: Supplemental data [file jciinsight-9-177229-s220.pdf]

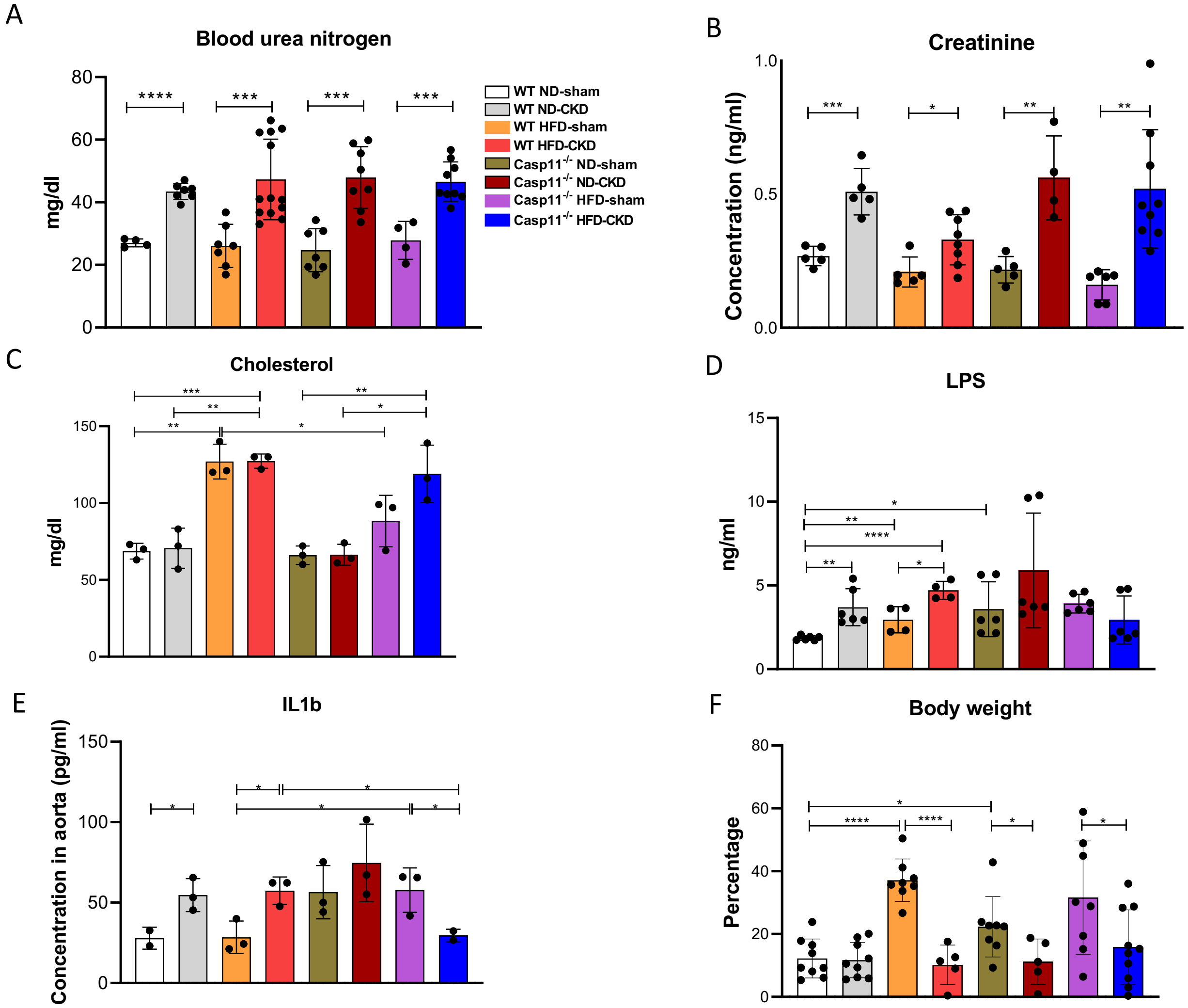

Supplementary Figure 1. A-C. The renal function markers blood urea nitrogen (**A**), creatinine (**B**), and cholesterol (**C**) levels were detected in the plasma of ND and HFD, WT and casp4/11$^{-/-}$, CKD and Sham mice. **D and E**. The LPS levels (**D**) and IL-1β secretion (**E**) were detected in the plasma of ND and HFD, WT and casp4/11$^{-/-}$, CKD and Sham mice using ELISA. **F**. The percentage of increased body weight from 9th week to 18th week versus body weight at 9th week was measured in ND and HFD, WT and casp4/11$^{-/-}$, CKD and Sham mice. *P<0.05, **P<0.01, ***P<0.001

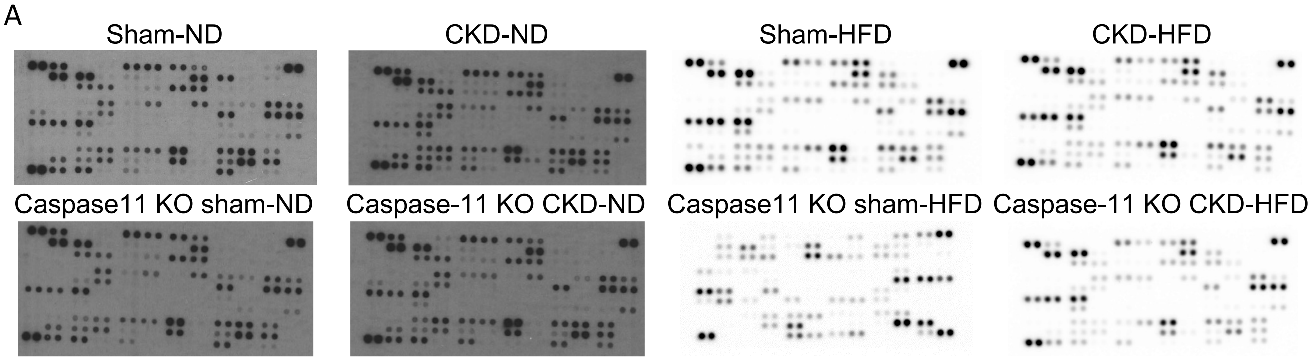

	1 2	3 4	5 6	7 8	9 10	11 12
A	RS	Adiponectin/Acrp 30	Amphiregulin	Angiopoietin-1	Angiopoietin-2	Angiopoietin-like 3
B		CCL6/C10	CCL11/Eotaxin	CCL12/MCP-5	CCL17/TARC	CCL19/MIP-3β
C		CD160	Chemerin	Chitinase 3-like 1	Coagulation Factor III/Tissue Factor	Complement Component C5/C5a
D	CXCL9/MI G	CXCL10/IP-10	CXCL11/I-TAC	CXCL13/BLC/BCA -1	CXCL16	Cystatin C
E	FGF acidic	FGF-21	Flt-3 Ligand	Gas 6	G-CSF	GDF-15
F	IGFBP-3	IGFBP-5	IGFBP-6	IL-1α/IL-1F1	IL-1β/IL-1F2	IL-1ra/IL-1F3
G	IL-10	IL-11	IL-12 p40	IL-13	IL-15	IL-17A
H	Leptin	LIF	Lipocalin-2/NGAL	LIX	M-CSF	MMP-2
I	PDGF-BB	Pentraxin 2/SAP	Pentraxin 3/TSG-14	Periostin/OSF-2	Pref-1/DLK-1/FA1	Proliferin
J	RS	E-Selectin/CD62E	P-Selectin/CD62P	Serpin E1/PAI-1	Serpin F1/PEDF	Thrombopoietin

	13 14	15 16	17 18	19 20	21 22	23 24
A	BAFF/BLyS/TNFSF13B	C1q R1/CD93	CCL2/JE/MCP-1	CCL3/CCL4/MIP-1α/β	CCL5/RANTES	RS
B	CCL20/MIP-3α	CCL21/6Ckine	CCL22/MDC	CD14	CD40/TNFRSF 5	
C	Complement Factor D	C-Reactive Protein/CRP	CX3CL1/Fractalki ne	CXCL1/KC	CXCL2/MIP-2	
D	DKK-1	DPPIV/CD26	EGF	Endoglin/CD105	Endostatin	Fetuin A/AHSG
E	GM-CSF	HGF	ICAM-1/CD54	IFN-γ	IGFBP-1	IGFBP-2
F	IL-2	IL-3	IL-4	IL-5	IL-6	IL-7
G	IL-22	IL-23	IL-27 p28	IL-28A/B	IL-33	LDL R
H	MMP-3	MMP-9	Myeloperoxidase	Osteopontin (OPN)	Osteoprotegerin / TNFRSF11B	PD-ECGF/Thymidine phosphorylase
I	Proprotein Convertase 9/PCSK9	RAGE	RBP4	Reg3G	Resistin	
J	TIM-1/KIM-1/HAVCR	TNF-α	VCAM-1/CD106	VEGF	WISP-1/CCN4	Negative Control

Supplementary Figure 2. The expression of pro-inflammatory and anti-inflammatory cytokines and growth factors was examined in the plasma of ND and HFD, WT and caspase-4/11-/-, CKD and Sham mice. The plasma of mice is collected at the 18th week from groups of HFD-CKD, HFD-sham, ND-CKD and ND-sham. After that, the plasma levels of pro-inflammatory, chemokines, anti-inflammatory cytokines and growth factors are detected in these groups using cytokine array. The cytokine array film images of ND-Sham, ND-CKD, HFD-Sham, HFD-CKD in WT mice are indicated. The cytokines, chemokines, and growth factors examined are indicated. Each sample is pooled from 3 mice in each group (n = 3).

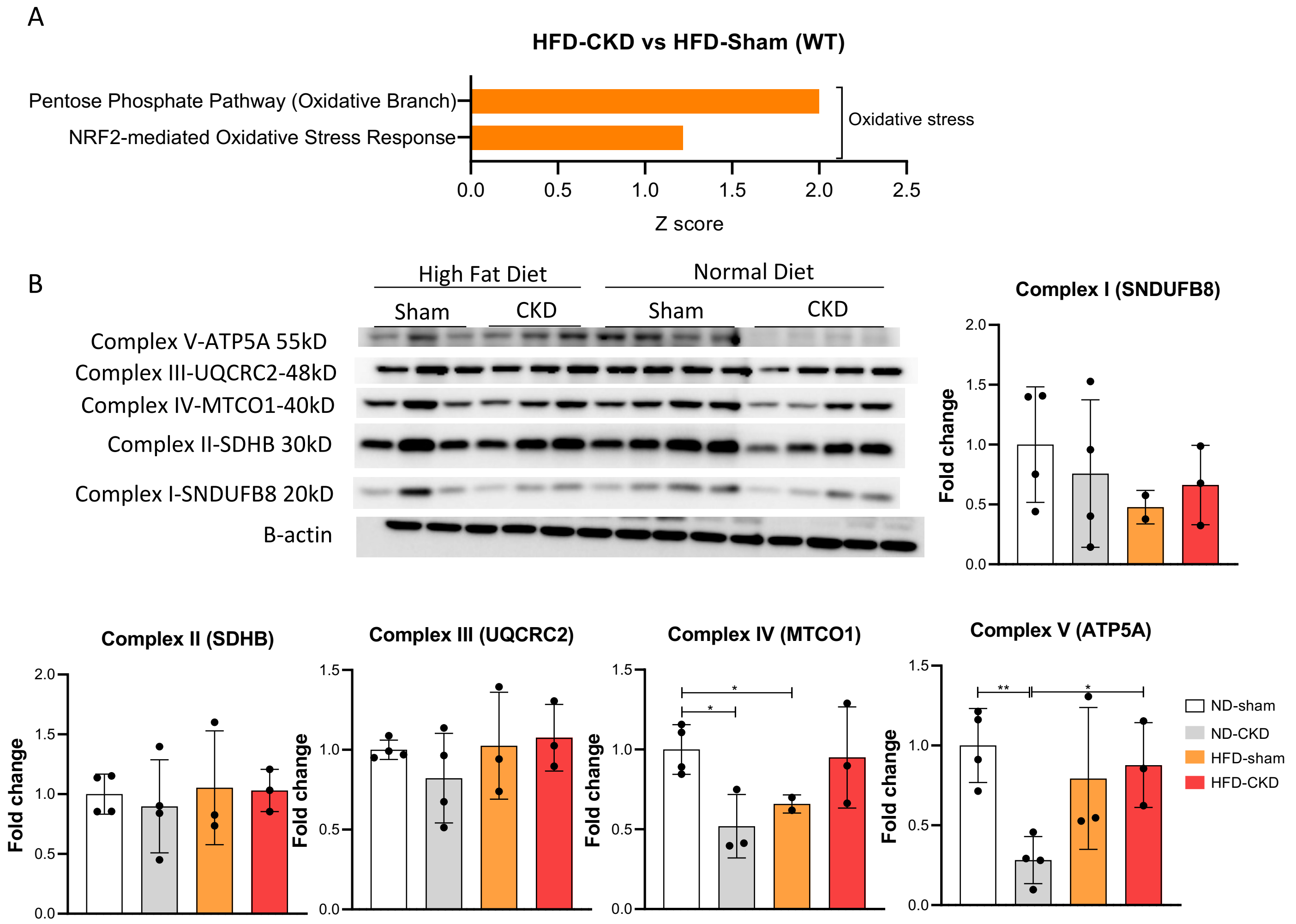

Supplementary Figure 3. ROS signaling is increased in the aorta of HFD+CKD. A. The oxidative stress pathways were analyzed in the aortic RNA-seq data from groups of HFD+CKD vs. HFD-sham. **B**. The expression of mitochondrial complexes was examined in the aortic lysates of HFD+CKD, HFD-sham, ND-CKD and ND-sham, and quantification of the expression of mitochondrial complex I (SNDUFB8), complex II (SDHB), complex III (UQCRC2), complex IV (MTCO1) and complex V (ATP5A) was quantified by ImageJ. * P<0.05, ** P<0.01.

A

GBP family gene

	HFD CKD vs HFD		HFD CKD vs CKD	
Gene	P	LogFC	p	LogFC
GBP2				
GBP3			0.01	0.88
GBP4				
GBP5				
GBP6	0.03	0.57	0.00	1.11
GBP7				
GBP10				
HMGB1	0.02	-0.33		
RAGE				

B

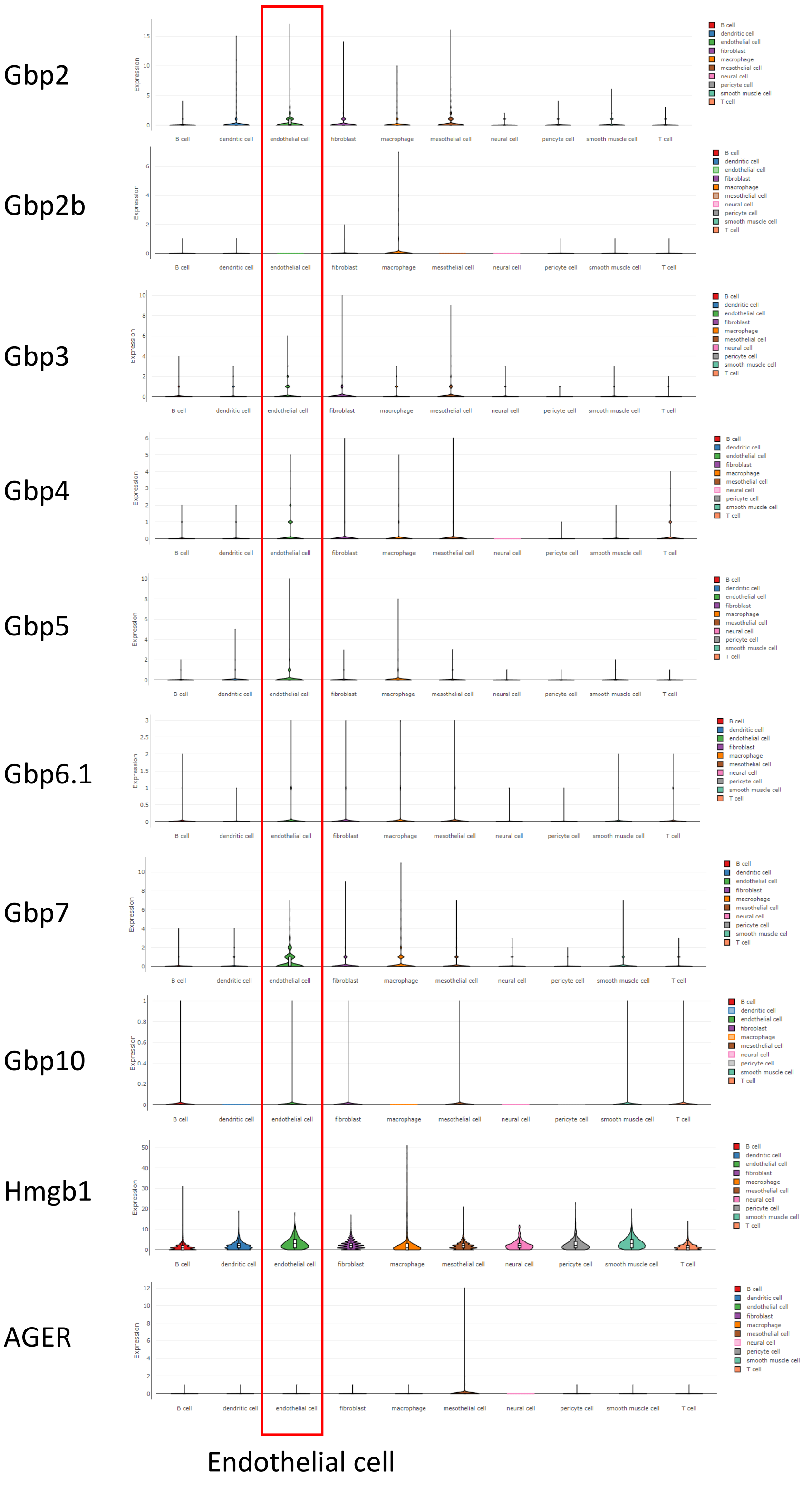

C

Canonical and non-canonical inflammasome regulator gene

HFD CKD vs HFD Canonical

Gene	P	LogFC
IL1B	0.03	1.96
ITPR2	0.00	1.07
CYBB	0.04	0.85
NAMPT	0.00	0.73
MAVS	0.00	0.66
TXN2	0.01	0.59

Non-canonical

Gene	P	LogFC
IL1B	0.03	1.96
SLC25A22	0.00	1.05
GBP6	0.03	0.57

HFD CKD vs CKD Canonical

Gene	P	LogFC
IL1B	0.02	2.10
ITPR2	0.00	1.32
MAVS	0.00	0.67
NAMPT	0.00	0.67
OAS2	0.03	0.66
TXN2	0.01	0.63

Non-canonical

Gene	P	LogFC
IL1B	0.02	2.10
SLC25A22	0.00	1.20
GBP6	0.00	1.11
GBP3	0.01	0.88

D

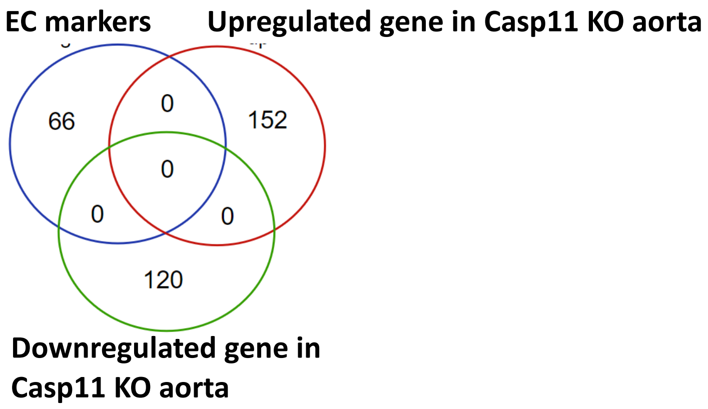

E

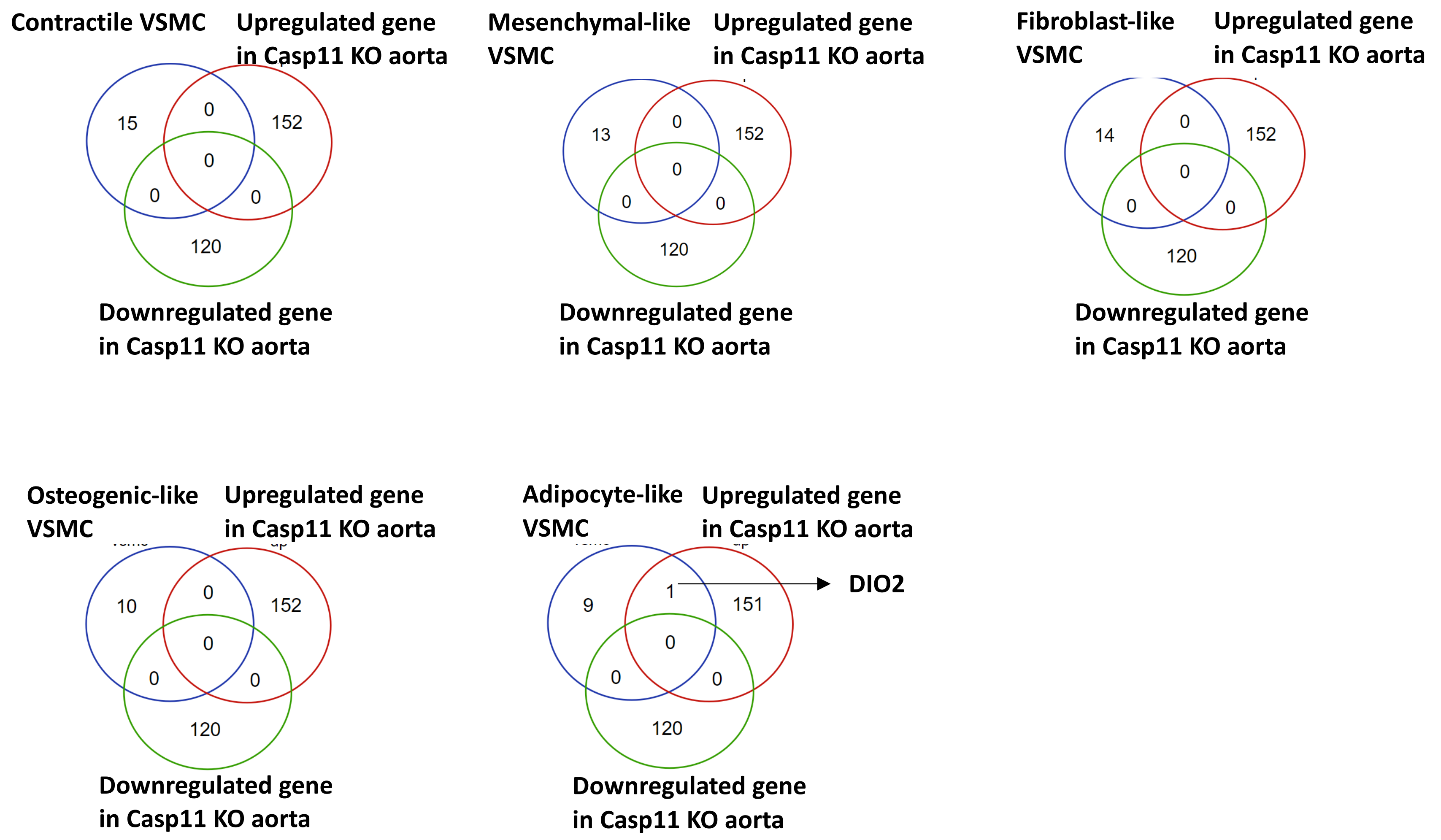

F

Potassium channel gene in CKD HFD vs. Sham ND

Gene	P	LogFC
KCNK2	0.02	2.41
KCNT1	0.04	-1.12
KCNK10	0.01	-2.15

Supplementary Figure 4. RNA-seq analysis in HFD+CKD vs. HFD and HFD+CKD vs. CKD.
A. The expression of GBP family genes in an RNA-Seq dataset. **B**. The single-cell RNA-seq (Single cell portal at the Broad Institute of MIT and Harvard) in the aortas of HFD fed mice showed upregulated LPS endocytic machinery components in endothelial cells . **C.** The expression of canonical and non-canonical inflammasome gene in an RNA-Seq dataset. **D.** EC marker gene list from PMID: 29333215, Upregulated and downregulated gene from Casp11 HFD CKD vs. WT HFD CKD group (P<0.05 and LogFC>1 or <-1) **E.** Vascular smooth muscle cell phenotype marker from PMID: 33324416; 34470477. Upregulated and downregulated gene from Casp11 HFD CKD vs. WT HFD CKD group (P<0.05 and LogFC>1 or <-1) **F.** 79 potassium channel genes collected from HGNC (https://www.genenames.org/data/genegroup/#!/group/183) are screened in RNA-seq of WT CKD HFD vs. WT Sham ND.

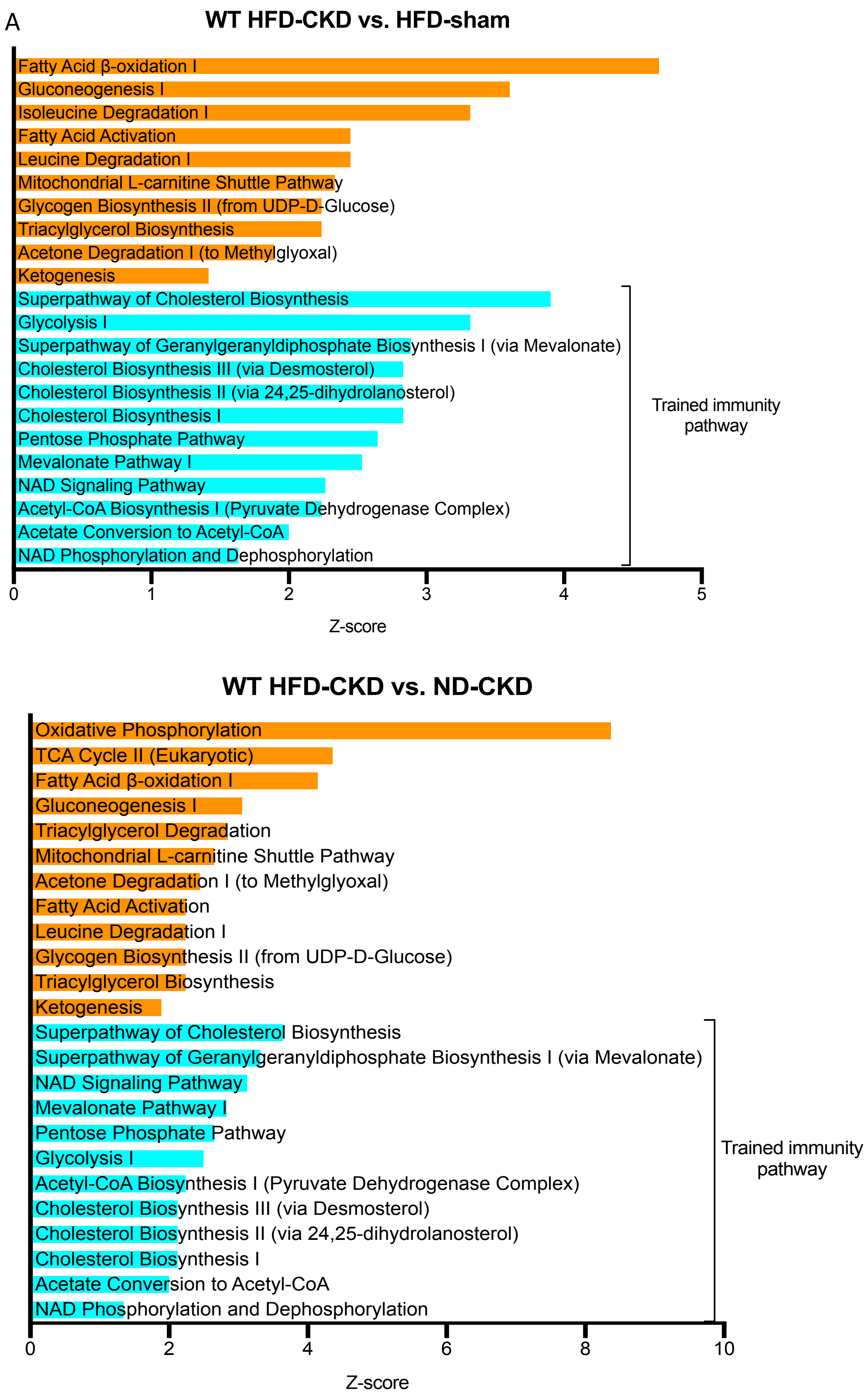

Supplementary Figure 5. the ingenuity pathway analysis (IPA) showed that HFD+CKD vs. HFD and HFD+CKD vs. CKD upregulated 12 TI pathways
