# Supplementary figures and images for: Caspase-4/11 promotes hyperlipidemia and chronic kidney disease–accelerated vascular inflammation by enhancing trained immunity
Source: JCI Insight. 2024 Jul 18;9(16):e177229. doi: 10.1172/jci.insight.177229 (PMC11343595; doi:10.1172/jci.insight.177229)

Figure 2H

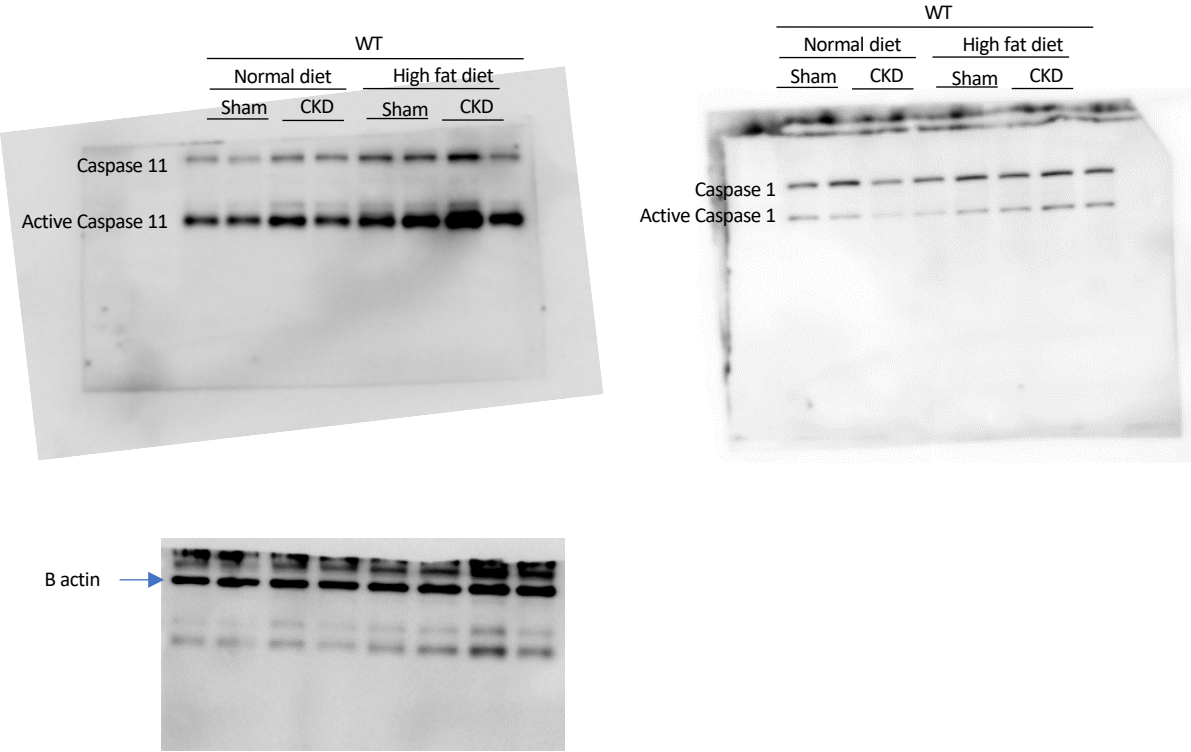

Figure 4E

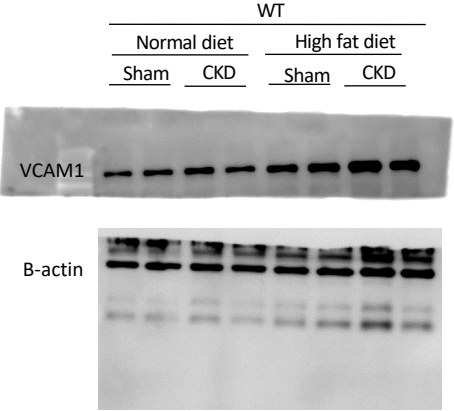

Supp. Fig 3B

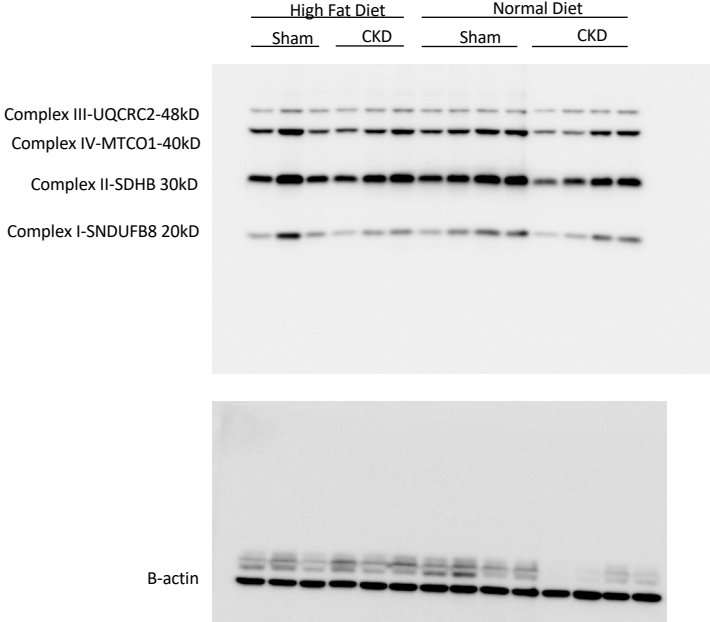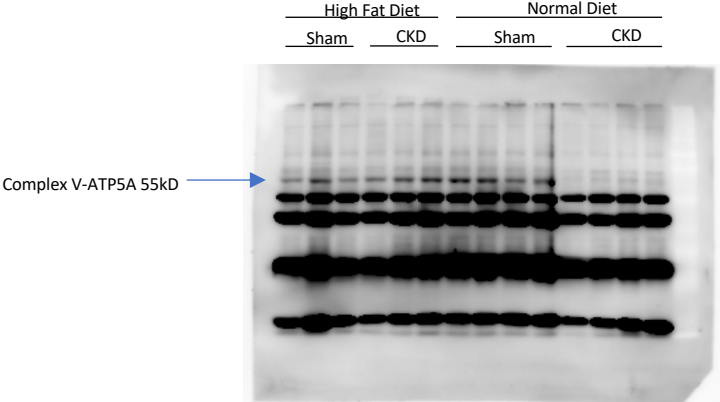

Supplement: Unedited blot and gel images [file jciinsight-9-177229-s221.pdf]
